# Metagenome-assembled genomes of phytoplankton microbiomes from the Arctic and Atlantic Oceans

**DOI:** 10.1186/s40168-022-01254-7

**Published:** 2022-04-28

**Authors:** Anthony Duncan, Kerrie Barry, Chris Daum, Emiley Eloe-Fadrosh, Simon Roux, Katrin Schmidt, Susannah G. Tringe, Klaus U. Valentin, Neha Varghese, Asaf Salamov, Igor V. Grigoriev, Richard M. Leggett, Vincent Moulton, Thomas Mock

**Affiliations:** 1 School of Computing Sciences, University of East Anglia, Norwich Research Park, Norwich, NR47TJ UK; 2 US Department of Energy Joint Genome Institute, 1 Cyclotron Road, Berkeley, CA 94720 USA; 3 School of Environmental Sciences, University of East Anglia, Norwich Research Park, Norwich, NR47TJ UK; 4 Alfred-Wegener Institute for Polar and Marine Research, Am Handelshafen 12, 27570 Bremerhaven, Germany; 5 Earlham Institute, Norwich Research Park, Norwich, NR4 7UG UK

## Abstract

**Background:**

Phytoplankton communities significantly contribute to global biogeochemical cycles of elements and underpin marine food webs. Although their uncultured genomic diversity has been estimated by planetary-scale metagenome sequencing and subsequent reconstruction of metagenome-assembled genomes (MAGs), this approach has yet to be applied for complex phytoplankton microbiomes from polar and non-polar oceans consisting of microbial eukaryotes and their associated prokaryotes.

**Results:**

Here, we have assembled MAGs from chlorophyll *a* maximum layers in the surface of the Arctic and Atlantic Oceans enriched for species associations (microbiomes) with a focus on pico- and nanophytoplankton and their associated heterotrophic prokaryotes. From 679 Gbp and estimated 50 million genes in total, we recovered 143 MAGs of medium to high quality. Although there was a strict demarcation between Arctic and Atlantic MAGs, adjacent sampling stations in each ocean had 51–88% MAGs in common with most species associations between *Prasinophytes* and *Proteobacteria*. Phylogenetic placement revealed eukaryotic MAGs to be more diverse in the Arctic whereas prokaryotic MAGs were more diverse in the Atlantic Ocean. Approximately 70% of protein families were shared between Arctic and Atlantic MAGs for both prokaryotes and eukaryotes. However, eukaryotic MAGs had more protein families unique to the Arctic whereas prokaryotic MAGs had more families unique to the Atlantic.

**Conclusion:**

Our study provides a genomic context to complex phytoplankton microbiomes to reveal that their community structure was likely driven by significant differences in environmental conditions between the polar Arctic and warm surface waters of the tropical and subtropical Atlantic Ocean.

**Video Abstract.**

**Supplementary Information:**

The online version contains supplementary material available at 10.1186/s40168-022-01254-7.

## Background

The global ocean arguably harbours the largest microbial diversity on planet Earth. To reveal insights into global marine microbial diversity, which is also considered to be the biogeochemical engine of our planet, multiple large-scale international projects, of which TARA Oceans [1] might be the most significant, have been conducted over the past 10 years. The outcome of these projects has provided a step change in our understanding of marine microbial diversity especially in the surface ocean. One of the most important revelations from these initiatives was the realisation that we have significantly underestimated plankton diversity in the past because we were too reliant on culture-dependent methods [2]. As a consequence, some of the groups we thought of as being insignificant in the oceans turned out to be highly diverse with a major contribution to the global carbon cycle and marine food webs [3]. Furthermore, the significance of organism interactions and specifically symbiosis for cycling of energy and matter was revealed [4, 5].

Linking functional microbial diversity with microbial activity as part of physico-chemical ecosystem properties sheds light on how different microbial groups contribute to biogeochemical cycling of elements [1, 6, 7]. These results built the foundation for estimating how changing oceans due to global warming might impact the diversity and activity of ocean microbes [7, 8]. However, to fully explore the role of microbes and their interactions in changing environmental conditions, we must understand their metabolic capabilities in a genomic context [9, 10]. As the majority of marine microbes are unculturable and because genomic information is required to reconstruct their metabolic evolution, metagenome-assembled genomes (MAGs) offer a solution [11, 12]. Although most MAGs are not at the level of quality achieved through sequencing cultures of isolated strains, they provide genome-level insights into the microbial diversity of natural ecosystems. Due to their small size and structural simplicity, bacterial and archaeal genomes have preferentially been assembled from metagenomes [13, 14]. Hence, the majority of published MAGs are of prokaryotic nature, and quite often eukaryotes are not even part of the underlying metagenomes due to the selective filtration of microbial communities.

To the best of our knowledge, there are less than 20 reports on MAGs from oceanic habitats, and most of them report on prokaryotic genome reconstructions [14–18]. However, Delmont et al. [19] assembled more than 700 eukaryotic genomes from *Tara Oceans* samples. These genomes represent a new genomic resource and will help to analyse metagenome and metatranscriptome datasets as their analysis is largely limited by the availability of reference genomes. This lack of reference genomes is particularly pronounced for eukaryotic microbes [20]. In addition to this phylogenetic bias, MAGs are also geographically biassed because most of them have been reconstructed from tropical and temperate oceans [14]. Recently, Delmont et al. [19] assembled some eukaryotic MAGs from the Arctic, and a recent metagenomics study in the Arctic and Southern Oceans retrieved 214 prokaryotic MAGs [21]. Hence, both papers appear to represent the first studies of this kind in polar seawater. This study, however, focusses on the eukaryote-enriched (size range 1.2–100 μm) phytoplankton microbiomes from polar and non-polar oceans. The reason for selective filtration was twofold: (1) to exclude small multicellular animals with relatively large genomes (e.g. copepods [22, 23]), which therefore would have dominated the metagenomes, and (2) to enrich for species associations (microbiomes) with focus on pico- and nanophytoplankton and their associated heterotrophic bacteria. We focussed on pico- and nanoplankton because they are well known to account for a significant proportion of marine primary production including polar oceans such as the Arctic Ocean [24–26]. Although these communities significantly contribute to the global carbon cycle especially in polar oceans [27–29], MAGs describing their uncultured genomic diversity including species associations have not been reported yet [1, 19].

Unlike in tropical and warm temperate oceans, primary production in polar oceans is mainly based on photosynthetic microbial eukaryotes such as diatoms, haptophytes, chlorophytes, and prasinophytes and their associated bacteria [30–32]. Many of the latter are part of the phycosphere, a microscale mucus region that is rich in organic matter surrounding a phytoplankton cell analogous to the rhizosphere in plants [33, 34]. Thus, organic matter released by phytoplankton is used as substrates for prokaryotes. Sometimes, these inter-kingdom species associations can be mutualistic with bacteria providing essential compounds such as vitamin B12. Although these phytoplankton microbiomes underpin some of the largest food webs on Earth, our understanding of their uncultured genomic diversity is very limited. This particularly is the case for polar regions and in a comparative context with their non-polar counterparts. The reasons are manifold but often driven by limited access to polar regions and the genomic complexity of eukaryotic phytoplankton [35, 36]. A majority of ocean microbes are not amenable to cultivation [37], so in order to increase the currently sparse set of genomes available for eukaryotic phytoplankton, alternatives to culture-based methods are necessary. Metagenomic sequencing offers the potential of insight into the taxonomic and functional composition of communities sampled, in addition to the generation of a genomic context for abundant organisms within the community through metagenomic binning approaches [38]. Comparative analyses of polar vs non-polar MAGs from uncultured microbes has only been reported a few times at least to the best of our knowledge [21], and we are not aware of any study addressing inter-kingdom species associations in the surface ocean based on MAGs. To address this knowledge gap, we selected the Atlantic and adjacent Arctic Ocean for sequencing eleven surface ocean metagenomes from chlorophyll *a* maximum layers enriched (size range 1.2–100 μm) for species associations (microbiomes) with a focus on pico- and nanophytoplankton and their associated heterotrophic bacteria. A total of 679 Gbp representing 4.53 billion reads from 6 Arctic and 5 North Atlantic metagenomes resulted in the recovery of 143 MAGs including several draft genomes of microalgae and their associated bacteria. A comparative analysis of all MAGs revealed polar-specific metabolism and a strong demarcation between MAGs from the Arctic vs tropical and subtropical Atlantic surface waters. Thus, our study provides novel insights into the uncultured genomic diversity of phytoplankton microbiomes from the relatively underexplored Arctic Ocean including the differences to their non-polar counterparts.

## Methods

### Sampling, DNA extraction and purification, sequencing, and taxonomic identification

Samples were collected on two RV Polarstern (Alfred-Wegener Institute for Polar and Marine Research, Bremerhaven, Germany) expeditions described by Martin et al. [39]. Eleven samples were taken from chlorophyll *a* maximum layer of the surface ocean for metagenome sequencing. Details of these stations along with environmental metadata are included in Additional file 1. Six of these were stations within the Arctic Circle and five in the tropical and subtropical Atlantic. Arctic samples were collected on ARK-XXVII/1 (PS80) between 17 June and 9 July 2012; Atlantic samples were collected on ANT-XXIX/1 (PS81) between 1 and 24 November 2012. After water samples were pre-filtered with a 100-μm mesh to remove bigger zooplankton, they were filtered onto 1.2-μm Nucleopore membrane filters and stored at − 80 °C until further analysis. DNA was extracted using the EasyDNA Kit (Invitrogen, Carlsbad, CA, USA) with some adjustments. Cells were washed off the filter with pre-heated (65 °C) solution A from the kit, and the supernatant was transferred into a new tube with one small spoon of glass beads (425–600 μm, acid-washed) (Sigma-Aldrich, USA). The samples were then vortexed three times in intervals of 3 s to break the cells. RNAse A was added to the samples and incubated for 30 min at 65 °C. The supernatant was transferred into a new tube, and solution B from the kit was added followed by a chloroform phase separation and an ethanol precipitation. DNA was pelleted by centrifugation and washed several times with isopropanol, air-dried, and suspended in 100 μL TE buffer. DNA concentration was measured with a Nanodrop (Thermo Fisher Scientific, Waltman, MA, USA), samples snap-frozen in liquid nitrogen and stored at − 80 °C until sequencing. Description of the samples and associated metadata is available through the GOLD database [40].

All eleven samples were sequenced and assembled by the Joint Genome Institute (JGI), while annotation was performed using the Integrated Microbial Genomes & Microbiomes (IMG/M) pipeline [41, 42]. In summary, paired-end sequencing was performed on an Illumina HiSeq platform. BBDuk (v35.87) [43] was used to remove Illumina adapters, then BBDuk filtering and trimming were applied. As part of the standard reads filtering and QC pipeline applied by JGI, reads mapping to the human HG19 genome with over 93% identity were discarded. The remaining reads were assembled with MEGAHIT (v1.0.3) [44]. The quality-controlled reads were mapped back to the assembly to generate coverage information using seal [45]. Some of these samples were later reassembled using SPAdes (v3.10.0-dev) [46]. For eukaryotic binning, we used only the MEGAHIT assemblies. Prokaryote bins come from either the MEGAHIT or SPAdes assembly for that sample, though no sample had both assemblies binned.

Genes were predicted by the IMG pipeline (v4.11-16) [42]. Briefly, genes were predicted from assembled contigs using prokaryotic GeneMark.hmm (v2.8), MetaGeneAnnotator (August 2008), Prodigal (v2.6.3), and FragGeneScan (v1.1.6) [47–50]; the number of copies of each gene is estimated from coverage of contigs generated by mapping back reads using seal [45]. Taxonomy was assigned to genes based on the top scoring USEARCH (6.0.294) result against an IMG reference database of non-redundant proteins from the isolate genomes. Contigs were assigned taxonomy of the last common ancestor of all genes on the contig, where more than 30% of genes have USEARCH hits. Where samples were later reassembled and annotated, as discussed in the previous paragraph, we use the estimated gene copies data from the most recent assembly.

### Binning

All contigs were binned by the IMG/M pipeline (v4.11-16) [41]. The bins generated from the complete set of contigs identified a number of bins, all of which were prokaryotic. Briefly, the binning process was that each assembly was binned separately, using MetaBat [51] and a minimum contig size of 3000 bp. The resulting bins were assessed for completeness and contamination with CheckM [52]. The taxonomy of bins was predicted using GTDB-Tk [53]. These prokaryotic MAGs are available on the IMG website using the bin identifiers available in Additional file 1. While eukaryotic sequences were not excluded from binning, all bins were labelled as archaea or bacteria. This lack of eukaryotic bins prompted the distinct binning attempt for eukaryotes.

For eukaryotic binning, each assembly was binned separately, the process for binning one assembly is given below. From all the station’s assembled contigs, eukaryotic contigs were predicted with EukRep (v0.6.5) [12], which uses a linear support vector machine to classify sequences as eukaryotic or prokaryotic using *k*-mer frequencies. The coverage of these eukaryotic contigs was estimated by pseudoaligning the reads from each sample to the contigs using Kallisto (v0.44.0) [54]. Binning was performed on only the eukaryotic contigs using MetaBat (v2.12.1) [51] with the coverage information and a minimum contig size of 1500 bp. This process identified 18 medium-quality MAGs [11].

After this initial binning effort for eukaryotes, the data was binned by different methods by partners at JGI. Briefly, taxonomic origins of contigs were determined using Mmseqs2 [55] searched against the NR and MMETSP [56] databases. Binning was again performed using Metabat. Bins with more than 50% of contigs assigned to a single eukaryotic phylum and a total length of greater than 5 Mb were considered potential eukaryotic MAGs and were filtered to remove contigs from other taxa. These methods identified an additional 3 medium quality MAGs.

Completeness and contamination of resulting bins were assessed with BUSCO (v3.0.2) [57], using the eukaryota_odb9 set of genes. Bins which were less than 50% complete were discarded from further analysis. We later reassessed the quality of these bins using EukCC (v0.2) [58], which selects a lineage appropriate set of markers. Completeness is defined as the percentage of expected single-copy genes from a selected gene set observed in a MAG, and contamination is defined as the percentage of single-copy genes observed in two or more copies.

Names have been assigned to MAGs composed of the station they were binned from, a numerical identifier, and a suffix of either P to indicate they are from the IMG prokaryotic binning, or E to indicate they are from the eukaryotic binning.

Contigs in all MAGs, both prokaryotic and eukaryotic, were concatenated and reads pseuodaligned back to this set of sequences representing all MAGs using Kallisto (v0.44.0) [54], to estimate the proportion of reads represented by the recovered MAGs.

### Phylogenetic placement

PhyloSift (v1.0.1) [59] was used to identify the sequences homologous to the mostly single copy genes in bins and reference genomes using the HMMs provided by PhyloSift. For eukaryotic reference genomes, all protists and green algae labelled representative from NCBI were used, as well as two diatom genomes (*Thalassiosira pseudonana*, *Phaeodactylum tricornutum*) taken from JGI. For prokaryotes, all genomes in the MarRef [60] database were included. Homologous sequences were located and the best hit retained when there were multiple. Viral marker genes were excluded. Marker genes present in less than 50% of the genomes (reference or MAGs) were not used in future steps of the analysis. Homologous sequences were aligned against the PhyloSift models, and alignments for all genes concatenated. RAxML (v8.2.12) [61] was used to build phylogenomic trees for the eukaryotic and prokaryotic alignments, using the GTRCAT model approximation with 100 bootstrap replicates. The resulting trees were visualised with Interactive Tree of Life Viewer [62] (Figs. 2 and 3).

We combined our *Bacillariophyta* MAGs with the non-redundant *Bacillariophyta* MAGs from Delmont et al. [19] along with 44 *Ochrophyta* reference genomes. This tree was constructed using genes located by BUSCO orthologous to the eukaryote_odb_10 gene set. Again, marker genes present in less than 50% of the genomes were excluded. Genes were individually aligned using MUSCLE [63], the alignments concatenated and trimmed using TrimAL’s -automated1 setting [64]. The tree was constructed with RaXML (v8.2.12) using the automatic model selection option PROTGAMMAAUTO with 100 bootstrap replicates (Fig. 7).

As additional evidence for taxonomy contigs from MAGs were searched against databases with BLAST (v2.9.0+) [65] and each contig assigned a taxonomy using the MEGAN-LR (v6) algorithm [66], eukaryotes were searched against the Marine Microbial Eukaryote Transcriptome Sequencing Project (MMETSP) [56], prokaryotes against NT. Selected groups of MAGs and reference genomes had ANI (Average Nucleotide Identity) calculated with pyani (v0.2.10) [67] using the BLAST-based ANIb method (Additional file 7). Average amino acid identity (AAI) was calculated using CompareM (v0.1.2) [68] between prokaryotic MAGs and proteins from the MarRef databse and between eukaryotic MAGs and algal proteins downloaded from PhycoCosm [69].

### Functional annotation

Functional annotation for contigs was carried out as part of the IMG/M pipeline before binning. Protein coding genes were predicted using an ensemble of prokaryotic gene prediction tools: Prodigal, prokaryotic GeneMark.hmm, FragGeneScan and MetageneAnnotator [47–50]. For prokaryotes, no further gene prediction and annotation were performed, and the annotations for the contigs before binning were used. Gene Ontology (GO) terms [70, 71] for genes were generated via the mapping of Pfam to GO terms maintained by the Interpro team [72].

The gene prediction tools used as part of the IMG/M pipeline are intended for prokaryotic gene prediction, so for eukaryotes, a further round of gene prediction was performed. The contigs in the initial 18 eukaryotic MAGs were predicted ab initio using the eukaryote-specific gene prediction tool GeneMark-ES (v4.38) [73] in self-training mode with MAKER (v2) [74]. GeneMark-ES assumes all sequences are from a single origin, so necessitated performing eukaryotic gene prediction after binning. The additional 3 MAGs later identified had genes predicted using MetaEuk [75] and the NR [76] database. In both cases, predicted proteins were annotated using InterproScan 5 (v5.37-75.0) [77] combining analyses from the following member databases: TIGRFAM (v15.0) [78], SFLD (v4) [79], SUPERFAMILY (v1.75) [80], Gene3D (v4.2.0) [81], Hamap (v2019_01) [82], ProSiteProfiles (v2019_01) [83], Pfam (v32.0) [84], MobiDBLite (v2.0) [85] and PIRSF (v3.02) [86].

### Inter-kingdom species associations and coverage

Associations between eukaryotic and prokaryotic MAGs were investigated by linear regression, using an ordinary least square regression method based on the mean coverage of each MAG. We kept any associations with *R*^2^ greater than 0.7 and a *p*-value of ≤ 0.05. Visual inspection suggested some correlations may be driven by single highly influential observations. We discarded any pairs which did not meet the criteria mentioned earlier when points with Cook’s distance greater than 1.25 were removed. The functions of one pair of associated MAGs (NP2_2E and NP3_22P) was examined. For each MAG, Fisher’s exact test was used to identify GO terms which are more commonly found in the selected MAG compared to a background set of MAGs. We classified the Eukaryote NP2_2E as a *Bathycoccus*, so used all *Prasinophyte* MAGs not involved in any associations as a background set. Similarly, as NP3_22P was classified as *Alphaproteobacteria*, we used all *Alphaproteobacteria* MAGs not involved in associations. We considered any terms overrepresented in the associated MAG with *p* ≤ 0.05 to be enriched in the MAG. In addition, we looked at terms enriched in two controls sets of MAGs which were not involved in any associations.

Coverage for each MAG was generated by aligning reads from each sample back to the bins using Bowtie2 (v2.3.5.1) [87]. Detection and mean coverage were calculated from these alignments using BedTools (v2.29.2) [88]. We adopt the same criteria as [89] and considered a MAG present in a given sample if in the alignment of reads from that sample against the MAG, at least 50% of bases in the MAG were covered by one or more reads.

## Results

### Metagenome sequencing and annotation of contigs

**Fig. 1:**
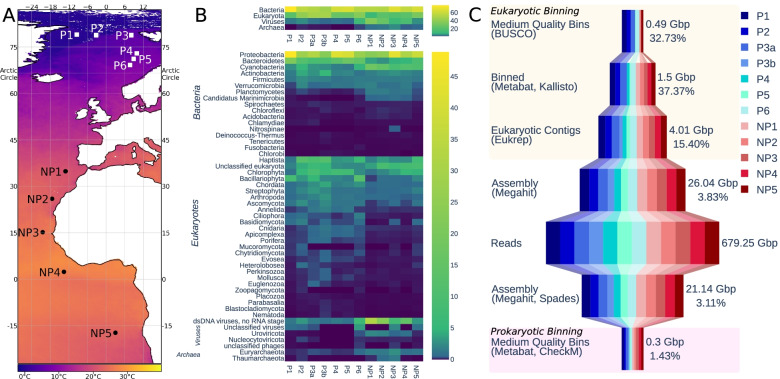
Summary of metagenomic sample, taxonomy, and binning. **A** Map showing the sampling locations. The horizontal black line shows the Arctic Circle. Colour indicates the mean annual sea surface temperature for the year of sampling [109]. **B** Relative estimated gene copies in each sample, based on genes predicted from assembled contigs and contig coverage. Top plot shows the data summarised to the rank of superkingdom; bottom is summarised to the rank of phylum. **C** Summary of the size of data at different points of processing. The pink box indicates the steps in the prokaryotic binning process, and peach those in eukaryotic binning. The number of bases is the size of data in this step, and the percentage is the percentage of the data retained from the previous step

Sampling stations have been named according to their geographical location in relation to the Arctic Circle. P-stations (polar) were located north and NP-stations (non-polar) south of the Arctic Circle in the North and South Atlantic (Fig. 1 a). In total, eleven stations were sampled (P1-6; NP1-5), and one metagenome was generated per station except for P3, which was used to sequence two metagenomes from two independent samples obtained from the chlorophyll *a* maximum layer. These two samples were labelled P3a and P3b. Sequencing all samples resulted in 4.53 billion reads totalling 679.25 Gbp, with each sample ranging between 46.79 and 67.37 Gbp. Assembling each station with MEGAHIT resulted in 42.10 million contigs totalling 23.02 Gbp.

IMG/M predicted 50.30 million genes in all sequenced metagenomes. Domains homologous to those in the Pfam database were found in 13.83 million (27.51%) of the predicted genes. Within samples, this proportion varied from 17.97 to 33%. The two samples from P3 had the lowest ratio of genes with homologous Pfam domains, both under 20%. The estimated counts of Pfam domains and GO terms for each sample are available in Additional file 9. Taxonomic affiliations were assigned to 17.74 million genes, of which 66% prokaryotic, 28% were eukaryotic, and 6% viral. The percentage of estimated gene copies in each sample is shown in Fig. 1 b at the level of superkingdom and phylum. The most abundant genes were of bacterial origin followed by eukaryotes, viruses and archaea. On the phylum level, genes from *Proteobacteria* were most abundant with *Haptista* being the most abundant eukaryotic phylum followed by *Chlorophyta*. Generally, eukaryotes are more abundant in polar stations, contributing between 25 and 46% of the total genes, whereas they only contribute between 10 and 31% in non-polar stations. In non-polar stations with a lower abundance of eukaryotes, there is a corresponding increase in the abundance of archaea and viruses. Differences between the means of gene counts between polar and non-polar stations are statistically significant for eukaryotes, viruses and archaea (Additional File 8), assessed using a *t*-test at a significance level set at ≤ 0.05. Photosynthetic eukaryotes such as *Chlorophytes* and *Bacillariophytes* generally have higher relative abundance in polar stations, whereas *Cyanobacteria* are more abundant in non-polar stations based on the relative contribution of genes.

A majority (93.5%) of the identified GO terms were shared between polar and non-polar samples. However, the proportion of Pfam domains with an unknown function was higher for domains uniquely found in either polar or non-polar stations than shared between them. Domains of unknown function constitute 16.55% of shared domains, but 23.76% and 29.71% in polar and non-polar metagenomes, respectively. Among domains unique to polar samples, 63.57% were identified in only one sample, and none was in all samples. For non-polar samples, only 43% of domains were present in only one sample, and 8.50% were in all samples.

### Metagenome-assembled genomes (MAGs)

#### Binning and quality

Metagenome binning generated 143 MAGs of medium and high quality, following the definitions for quality in [11]. Medium quality requires a completeness of at least 50% and contamination less than 10% and high quality a completeness of greater than 90% and contamination less than 5%, as well the presence of certain rRNA genes and tRNAs. These MAGs represent 0.71 Gbp of assembled reads (Fig. 1 c), while 8.1% of all reads mapped back to the sequences contained in the combined 143 MAGs. Of all bins, 21 were eukaryotes, 116 were classified as bacteria and 6 were archaea based on GTDB-Tk classification [53]. Slightly more prokaryotic MAGs were retrieved from non-polar than polar metagenomes, 64 and 58, respectively. The low number of archaea MAGs recovered fits with their low abundance based on the estimated number of gene copies, ranging from 0.24 to 4.93% of reads across samples. All prokaryotic MAGs from polar samples were classified to at least the phylum as either *Bacteroidota*, *Proteobacteria* or *Verrucomicrobiota*. Prokaryotic MAGs from non-polar metagenomes were more diverse and included the 6 archaea. These non-polar MAGs included phyla for which no polar MAGs were recovered: 6 *Actinobacteriota*, 8 *Myxococcota*, 2 *Patescibacteria*, 5 *Planctomycetota* and 1 *Poribacteria*.

Filtering the assembly for each sample to retain only eukaryotic contigs as predicted by EukRep resulted in 2,151,309 contigs totalling 4.01 Gbp. From these, we recovered 21 medium-quality eukaryotic MAGs. Only four of these eukaryotic MAGs were retrieved from non-polar metagenomes, which is congruent with the lower abundance of eukaryotes observed in the taxonomic assignment of genes prior to binning, as shown in Fig. 1b. Taxonomy was assigned to the eukaryotic MAGs based on their placement in a phylogenomic tree; 8 placed with *Mamiellophyceae* reference genomes and 10 with *Bacillariophyta*, and the placement of the remaining 3 was less clear. All but one of the *Bacillariophyta* were recovered from polar metagenomes. Polar *Mamiellophyceae* MAGs were placed in a clade with *Micromonas* and the non-polar MAGs with *Ostreococcus* or *Bathycoccus*.

Prokaryotic MAGs have a mean completeness of 74.30% and contamination of 2.68%. The MAG with the highest completeness is P1_21P at 99.62% and a contamination of 2.81%. Taxonomically, this MAG was classified to the family level as *Flavobacteriaceae*. Prokaryotic MAGs have a median L50 of 11,402 bp and a median size of 2.23 mbp. Eukaroyotic MAGs have a mean completeness of 67.82% and contamination of 2.82% as estimated by EukCC, with a median size of 24.31 mbp. Details of the MAGs are available in Additional file 1. The MAG with the highest completeness is P2_1E at 92.97%. All but one MAG is fragmented, with a median L50 of 5229 bp. The exception is P2_1E, which contains many contigs longer than 50 kbp, the longest being 106 kbp.

Some phyla with relatively high abundance in the taxonomic classification of genes had no MAGs retrieved. *Haptista* contributed a large proportion of the genes but no MAG could be confidently identified as *Haptista*, whereas MAGs were retrieved for the less abundant *Bacillariophyta* and *Verrucomicrobiota*.

#### Phylogenomic placement

##### Prokaryotes

**Fig. 2:**
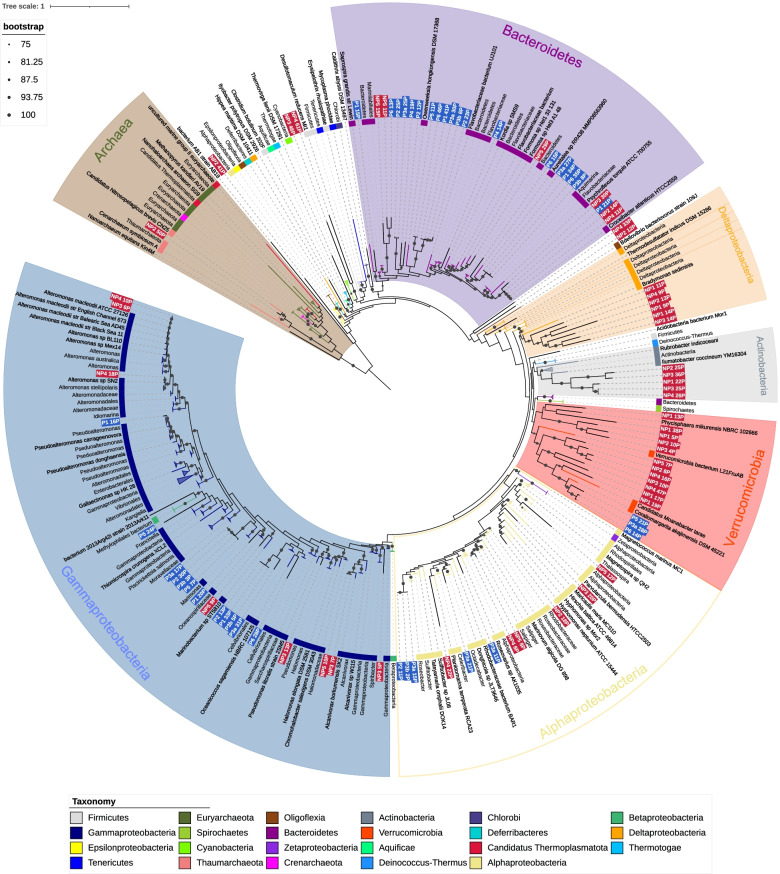
Phylogenomic tree for prokaryotic MAGs and reference genomes. Phylogenomic tree including prokaryotic MAGs and MarRef reference genomes. Inner band colour indicates taxonomy of reference genomes, using the NCBI taxonomy. MAG labels have a blue background for polar MAGS and a red background for non-polar. Clades which contained reference genomes all from the same taxonomic group in the legend have been collapsed; the size of triangle is scaled to the number of leaves in the collapsed clade. Collapsed clades have been given labels which encompass all the contained leaves. Bootstrap values are indicated by grey dots on branches

The phylogenomic tree for prokaryotes in Fig. 2 was constructed using concatenated alignments of 38 marker genes, a subset of those included in the PhyloSift package. Genomes of marine prokaryotes were retrieved from the MarRef database, for a total of 943 reference genomes (Suppl. Data 4) in addition to the 122 prokaryotic MAGs recovered in our study. The tree includes MAGs in which 50% or more of the selected marker genes were identified, a total of 88 of the MAGs. The largest group consists of 31 MAGs which placed within a clade with alpha-, beta- and *Gammaproteobacteria* references. A further 24 were placed with *Bacteroidota*.

At the level of phylum, the phylogenomic tree is largely in agreement with the taxonomies predicted by GTDB-Tk. There are some instances where MAGs have not been placed close to any of the included references, such as NP34_33P and NP2_12P, where GTDB suggested a more specific estimate; NP2_12P was assigned to a class of *Poribacteria*, for which no reference genomes are included in the MarRef data.

Some MAGs recovered from different stations appear closely related to one another. NP4_10P and NP3_6P are closely related to each other as well as to multiple *Alteromonas macleodii* strains. The reference genomes for *A. macleodii* can be split into those from surface and deep ocean [90]; these MAGs have a greater than 95% ANI to three surface genomes, suggesting a species-level relationship. The ANI between these MAGs and deep ocean *A. macleodii* is below 95%. This is supported by the assignment of contigs within the MAGs based on BLAST searches against the NT database, for both MAGs at least 89% of contigs are assigned to the *A. macleodii* node or a strain below it.

Other groups of MAGs display similarly close relationships to each other but are more distant from reference genomes. Four polar MAGs which placed among *Bacteroidetes*, P6_35P, P3b_8P, P1_34P and P3a_27P, share over 95% identity to each other but less than that to their closest reference genome, an unclassified species of genus *Aureitalea*. The results of assigning contigs via BLAST searches are similarly mixed, most contigs being assigned to a mix of *Flavobacterieaceae* or uncultured bacterium. These four MAGs could represent members of the same novel species of *Bacteroidetes*.

There are few close relationships between polar and non-polar MAGs evident in the tree. The median distance from a polar MAG to the nearest polar MAG is lower than to the nearest non-polar MAG and the same for non-polar to non-polar (Additional file 5). In both cases, the difference in the medians is significantly different at *p* < 0.01 using Mood’s median test. One clade of *Bacteroidetes* is an exception, where polar MAG P1_21P appears closely related to NP2_14P, NP3_30P and NP4_11P. The closest reference is *Croecibacter atlanticus* which is in a different clade. Pairwise ANI between these MAGs and the *C. atlanticus* reference genome is greater than 95%, suggesting these MAGs could represent genomes of the species *C. atlanticus*.

Some MAGs had been classified at a genus level by GTDB-Tk and species level by CheckM, where the length of branches in the phylogenomic tree do not suggest as close a relationship. MAGs P3a_28P, P6_14P, P5_21P, P2_21P and P6_33P were classified within the genus *Puniceicoccaceae*. The first three were placed closest to *C. akajimnesis* but with longer branches than observed between taxa from the same species elsewhere in the tree. The latter two lacked the amount of marker genes required to be included in the tree. Looking at the ANI also suggests these MAGs and *C. akajimensis* are not the same species, no pair shares above 95% ANI.

##### Eukaryotes

**Fig. 3:**
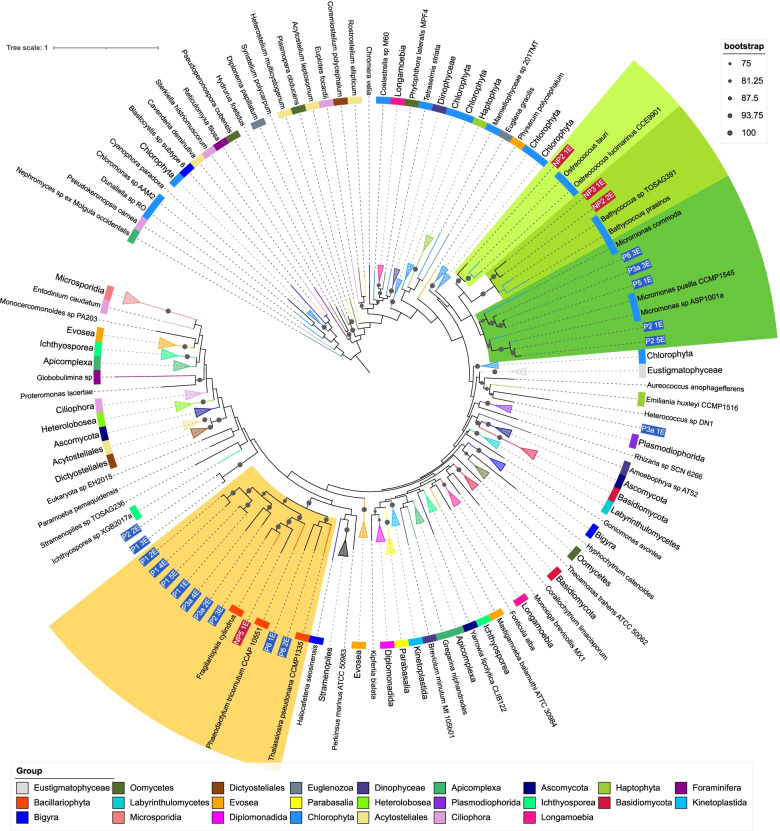
Phylogenomic tree for eukaryotic MAGs and reference genomes. Phylogenomic tree including eukaryotic MAGs and reference genomes. Label and inner band colour indicate taxonomy of reference genomes, using the NCBI taxonomy. MAG labels have a blue background for polar MAGS and a red background for non-polar. Clades which contained reference genomes all from the same taxonomic group in the legend have been collapsed. Coloured ranges highlight clades where MAGs place with reference genomes of a consistent taxonomy. Bootstrap values are indicated by grey dots on branches

The phylogenomic tree for eukaryotes in Fig. 3 was constructed using concatenated alignments of 57 marker genes, a subset of those included in the PhyloSift package. Representative genomes of microbial eukaryotes were retrieved from the National Centre for Biotechnology Information (NCBI) and JGI, for a total of 412 reference genomes (Additional file 4) in addition to the 21 eukaryotic MAGs recovered in our study. Most MAGs are placed in three clades, which contain all of the *Bacillariophyta* or *Mamiellophyceae* reference genomes. As branches within these clades are long, a more specific identification of these MAGs is difficult because of a lack of a sufficient number of reference genomes from eukaryotic marine microbes. Within the *Mamiellophyceae* clade, three MAGs (P6_3E, P5_1E, P3a_3E) are closely related to one another, but relationships to the reference genomes are more distant. *Bacillariophyta*-like MAGs appear to have more distant relationships (Fig. 3). P2_2E and P1_3E are difficult to provide a taxonomy for. They were placed close to each other, but distant from any reference genomes, and searches against MMETSP had no results for over 90% of contigs and have low AAI identity to reference proteins (maximum ca. 52% to *Picocystis* sp. ML).

*Mamiellophyceae*-like MAGs appear to further divide into three clades containing reference genomes from the three genera *Micromonas*, *Bathycoccus* and *Ostreococcus*. *Micromonas* MAGs were only recovered from polar and *Bathycoccus* and *Ostreococcus* only from non-polar metagenomes. Some *Mircomonas* MAGs have high average nucleotide identity (ANI) to each other or to reference genomes. For instance, MAGs P2_1E and P2_4E have 99% ANI with *Micromonas* sp. ASP10-01a, a species reconstructed from an Antarctic metagenome [83, 91]. Three MAGs appear highly similar: P6_3E, P5_1E and P3a_3E. ANI between these MAGs is 98% or higher and 99% between P5_1 and P3a_3. However, this group do not share high ANI with any of the reference genomes used. AAI supports the placements in the phylogenomic trees, for instance, NP2_1E is placed close to *Ostreococcus* references in the three and shows the highest AAI to *Ostreococcus lucmarinu*s (73.46%).

There is consistency in the taxonomic assignments of contigs within *Mamiellophyceae* MAGs at the phylum level. With the exception of NP2_1E, they have over 99% of their contigs assigned to *Chlorophyta* when searched against MMETSP as explained in the “Methods” section. The contigs that were not assigned to *Chlorophyta* were either assigned to the *Eukaryota* node, or had no BLAST hits. No contigs were assigned to other phyla. This suggests a consistent taxonomic origin for the sequences in these MAGs at least at the phylum level, rather than representing sequences which are not biologically related. Evidence from these BLAST searches supports the taxonomies suggested by the phylogenomic tree at the genus level; all *Mamiellophyceae* MAGs had at least 87% of their contigs assigned to the genus they placed within the phylogenomic tree.

Within NP2_1E, there is less confirmatory evidence in the results of the BLAST searches, a greater number of contigs are not assigned a taxonomy or assigned to other phyla. This could represent either a MAG for an organism more distantly related to sequences available in the reference database, or increased contamination within the MAG. Contigs with no BLAST hits contributed 34.12% of all contigs. For those contigs that did have hits, 96% were assigned to *Chlorophyta*, which represents 63.44% of the total contigs in the MAG. Contigs assigned to other phyla constitute 2.13% of the total.

Eight MAGs were placed in a clade with *Bacillariophyta* reference genomes, only one of which was non-polar. None of the MAGs appears close to the three reference genomes used in the phylogenomic tree. Some *Bacillariophyta* MAGs could be classified at the genus level. For instance, MAG P2_3E had an ANI of 85.5% and 83.15% AAI to *Fragilariopsis cynlindrus*, supporting their close placement. The next highest AAI among *Bacillariophyta* MAGs is much lower, 66.99% between NP5_1E and *Pseudo-nitzschia multiseries.* MMETSP contains sequences from *Bacillariophyta* taxa which currently lack a complete genome, results from searching sequences in that MAGs against this database provided further evidence for taxonomy. Apart from MAG P3a_4E, all the MAGs in the *Bacillariophyta* clade had 85% or more of their assigned contigs classified at the level of phylum when searched against MMETSP as described in the “Methods” section. P3a_4E had a majority of contigs assigned to *Bolidophyceae*, a sister taxa to *Bacillariophyta*. Additional close placement was obtained for P6_2E for which ca. 84% of contigs were classified as *Leptocylindrus danicus*, P3a_2E for which ca. 96.14% of contigs were classified as *Minutocellus polymorphus* and P1_5E for which ca. 85% of contigs were classified as *Chaetoceros neogracilis.* P1_1E shows a high ANI to our potential *Chaetoceros* MAG P1_5E; however, a lower proportion of contigs in P1_1E (ca. 69%) were assigned to *Chaetoceros*.

Many contigs in *Bacillariophyta* MAGs had no hits when searched against MMETSP with BLAST; a mean of 37.38% of contigs in *Bacillariophyta* MAGs had no hits. For comparison, the mean percentage of contigs in *Mamiellophyceae* MAGs which had no hits in MMETSP was much lower, at 4.45%.

The MAG P3a_1E placed closest (ca. 73% ANI) to the *Haptophyta*, *Emiliania huxleyi*. *E. huxleyi* is quite distant in the tree from the other two *Haptophyta*, *Chrysocromulina parva* and *Chrysocromulina* sp. CCMP2291, which are from the *Prymnesiales* order. These two *Prymnesiales* were placed as neighbouring leaves and showed 97% ANI. *E. huxleyi* and P3a_1 have much lower ANI with each other and the two *Prymnesiales* genomes. This MAG showed the highest AAI with a group of *Haptophyta* including *Phaeocystis* and *Chrysochromulina* species, with the highest being 62.59% AAI with *Phaeocystis antarctica*. Searching contigs from P3a_1E against MMETSP, a majority of contigs with hits were assigned to a range of *Haptophyta* taxa which included *E. huxleyi* among them, with most being assigned to *Phaeocystis antarctica*. Contigs were also assigned to several other phyla as well, possibly due to MAG contamination.

#### Inter-kingdom species associations and biogeographical distribution of MAGs

##### Inter-kingdom species associations

In total, we found 17 inter-kingdom species associations (15-positive and 2-negative associations) between MAGs from eukaryotic phytoplankton and heterotrophic bacteria based on correlating read coverage (*R*^2^ ≥ 0.7; *p*-value ≤ 0.05). Figure 6 shows three strong (*R*^2^ ≥ 0.79) and positive inter-kingdom associations between different *Mamiellophyceae* MAGs (*Bathycoccus* sp. NP2_2E/NP3_1E; *Ostreococcus* sp. NP2_1E) and Proteobacteria MAGs (*Erythrobacter* NP3_22P/NP322P; *Alteromonas* NP4_18P). These MAGs are widespread among the non-polar samples, the prokaryotes are observed in all non-polar samples and the eukaryotes in all but the southernmost NP5. Weaker positive associations (Additional file 8) were observed for three *Micromonas* MAGs (P3a_3E, P5_1E, P6_3E) and different *Gammaproteobacteria*, *Flavobacteriaceae* and *Puniceicoccaceae* MAGs. While these associations show high (*R*^2^ ≤ 0.97), they are driven by high coverage in the two samples P4 and P5 and low coverage elsewhere. A negative correlation was observed for a diatom MAG (P3a_2E) and *Colwellia* and *Porticoccaceae* MAGs, where the MAGs involved in the negative associations with *Bacilliarophyta* MAG P3a_2e have lower coverage across the polar samples.

Enriched GO terms for associated pair NP2_2E and NP3_22P is shown in Fig. 6 as an example. The only enriched cellular components in both MAGs were the membranes: the Golgi membrane for the *Bathycoccus* MAG and the outer membrane for the *Erythrobacter* MAG. Enriched molecular functions in the *Bathycoccus* MAG included glycosyltransferase activity and transport of pyrimidine nucleotide sugar. The *Erythrobacter* MAG was characterised by a more diverse number of molecular functions with several related to transmembrane transport, hydrolase, transferase and ligase activity.

Using the same method, we looked at the enrichment of MAGs which did not participate in associations as a control set (Additional file 10). We selected two pairs of eukaryote and prokaryote MAGs: one pair of *Prasinophyceae* and *Alphaproteobacteria* (P2_1E, P3a_15P) which are more closely related to the MAGS in Fig. 6, one pair of *Bacillariophyta* and *Gammaproteobacteria* (P3a_4E, NP3_6P) which are more distant. In the first control set, no terms were enriched in both the control pair and the associated pair; in the second more distantly related control set, only a single term of the 82 is enriched in the associated and control eukaryote MAG, where 11 of 92 shared by the prokaryotes.

##### Biogeographic distribution of MAGs

**Fig. 4:**
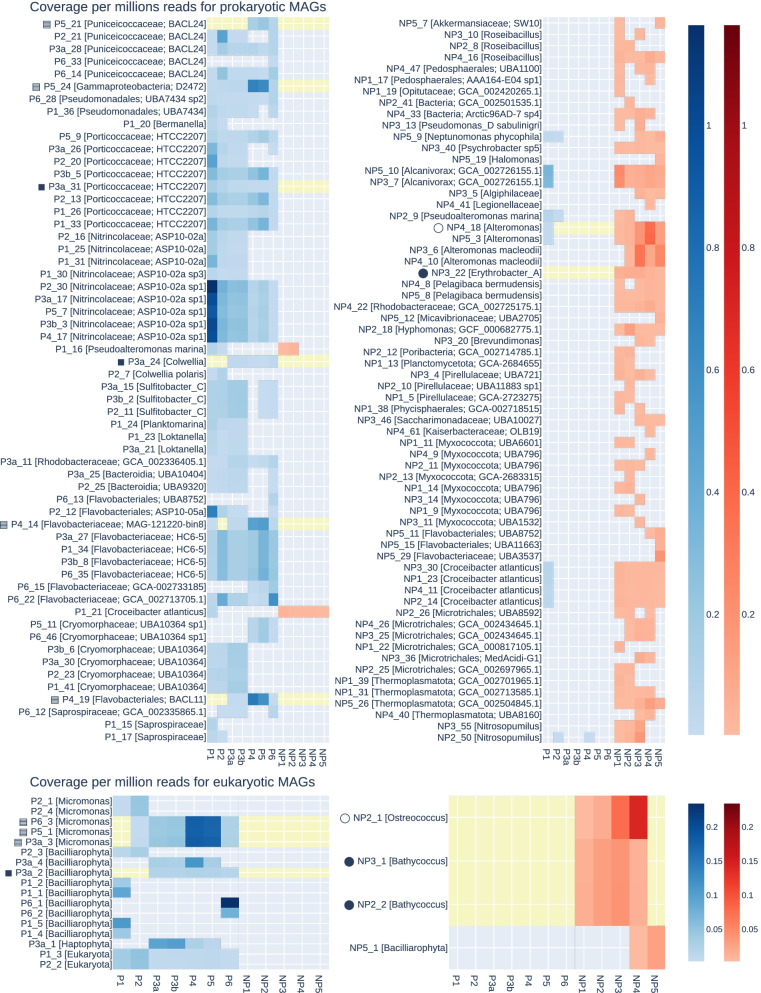
Coverage of MAGs across stations. The mean coverage of each MAG in a given set of reads. Top shows prokaryotic MAGs, and bottom shows eukaryotic MAGs. Coverage normalised to coverage per million reads. Coverage not shown where fewer than 50% of bases in a MAG had any read aligned. The left-hand heatmaps show MAGs recovered from polar assemblies, and right-hand heatmaps shows those recovered from non-polar assemblies. Coverage in reads from polar stations is shown on a blue scale, and coverage in non-polar stations is shown on a red scale. MAGs are ordered by taxonomy. Each MAG has been given a taxonomic label of the most specific rank to which taxonomy had been determined. MAGs involved in cross-kingdom associations are highlighted by a yellow band and with a shape (circle for non-polar or square for polar) to the left of their name. The shading of the shape indicates which MAGs are associated, for example, each eukaryotic MAG with a solid square has an association with each prokaryote with a solid square

Read coverage was also used to analyse MAG distribution across polar and non-polar samples. Where less than 50% of bases had at least one read aligned to them, we considered a MAG to not be present at that station [89]. The mean coverage of those prokaryotic MAGs present ranged between 0.89 and 375.07 with a mean coverage of 35.10. We used the mean coverage per million reads as an estimate of abundance of MAGs across stations (Fig. 4).

The binning process uses covarying coverage to group contigs into bins. Thus, for highly similar MAGs recovered from different assemblies, a similar pattern of coverage across sites would be expected. Four proteobacteria MAGs which appeared closely related in the phylogenomic tree, P3a_17P, P2_30P, P3b_3P and P5_7P, show this pattern strongly, with very similar patterns of changing coverage from stations P1 to P6. The coverage of MAGs tends to form a gradient across stations with close geographic proximity. For the most part, there is a clear demarcation between polar and non-polar MAGs. Of the 122 prokaryotic MAGs, 116 are only present in either polar or non-polar samples. MAGs detected in both tend to be detected in samples P1 and P2.

For eukaryotic MAGs present in a sample, the mean coverage ranged between 0.92 and 87.24, with a mean lower than that of prokaryotes at 17.68 (Fig. 4). Again, highly similar *Micromonas* MAGs P6_3E, P5_1E and P3a show very similar pattern coverage from stations P2 to P6. The coverage of MAGs tends to form a gradient across stations with close geographic proximity. There is a clear demarcation between polar and non-polar eukaryotic MAGs, as no MAG was found to be on both sides of the Arctic Circle. This coverage is limited to describing only the distribution of those members of the community for which a MAG was recovered. There are phyla which appeared abundant such as *Haptista*, which no recovered MAG clearly belongs to, which have the potential to contain widespread species which would not follow the strong demarcation observed.

Approximately half of the *Bacillariophyta* MAGs were present at only one or two stations maximum whereas *Mamiellophyceae* MAGs were generally more widespread. The one non-polar *Bacillariophyta* MAG is present only in stations NP4 and NP5, the southernmost of the non-polar stations. Potential *Haptophyte* P3a_1E is present in three polar stations, and most abundant at P3, where the *Bacillariophyta* MAGs are less abundant.

In both prokaryotes and eukaryotes, the MAGs that could not be assigned a taxonomy, assigned either as unknown, bacteria or eukaryota, are mostly observed in three or fewer stations, with low coverage.

#### Functional annotation of all MAGs

A PCA analysis of the GO term abundance in each MAG (Fig. 6) largely shows separation into taxonomic groups, supporting the broad classifications drawn from the phylogenomic tree for eukaryotes and the classifications from GTDB-Tk for prokaryotes. Clustering by taxonomy is stronger for eukaryotes than prokaryotes. The two large groups of *Bacillariophyta* and *Mamiellophyceae* are clearly separated, with the possible *Bolidophyceae* P3a_4E closer to P3a_1E the potential *Haptophyte*. Some prokaryotic groups form clear clusters, such as *Bacteroidota* and *Verrucomicrobiota*, while others are more spread such as the *Proteobacteria*. The number of GO terms observed in these groups is shown by bars at the top of Fig. 6, for the whole population before binning and for eukaryotic and prokaryotic MAGs. The whole population showed a majority of GO terms were present in all studied geographical regions, suggesting a widely distributed shared core of functions. Among functions unique to either side of the Arctic Circle, prokaryotic MAGs had many more unique functions in non-polar waters whereas eukaryotes had more unique functions at polar waters. Only 4 eukaryotic MAGs had been recovered from non-polar metagenomes. This imbalance could partially explain the high number of functions unique to polar eukaryotic MAGs. Prokaryotic MAGs were more balanced across the Arctic Circle, 65 from non-polar and 58 from polar stations.

Terms related to cold exposure are among the most abundant terms observed only in polar environments. Ice binding (GO:0050825) is unique to the polar eukaryotic MAGs. Ice binding proteins have been observed in a wide range of organisms across the biological kingdoms, including diatoms and marine bacteria [92]. The proteins encompass a range of activities; among polar algae, the recrystallisation inhibition has been suggested may maintain brine pockets which form during the freezing of seawater, providing a viable habitat in freezing conditions [93]. Among prokaryotes, the most abundant term unique to polar MAGs is heat shock protein binding (GO:0031072). Heat shock proteins were observed to be expressed in arctic *Rhizobium* species in response to heat stress [94] and in response to suboptimal temperatures in *Alicyclobacillus acidoterrestris* [95]. Terms related to photosynthetic activity in prokaryotes are unique to non-polar MAGs, with photosynthesis and photosystem II (GO:0015979, GO:0009523) among the most abundant unique terms.

Some differences appear driven by the taxonomy of MAGs recovered in the two areas. *Micromonas* have flagellum-based motility, and *Micromonas* MAGs were only recovered in polar samples. Consequently, some terms related to flagella such as cilium assembly (GO:0060271) which is considered equivalent to microtubule-based flagellum assembly. Some of the unique polar terms are driven by the two unidentified MAGs P2_2E and P1_3E, such as the most abundant unique polar term “homophilic cell adhesion via plasma membrane adhesion molecules” (GO:0007156), for which 95% of annotations were observed in genes from these unidentified MAGs.

## Discussion

### Binning and retrieving of MAGs from phytoplankton metagenomes

Sequences and MAGs from prokaryotes dominated as they were likely more abundant as commonly observed in phytoplankton microbiomes (Fig. 1) [33, 96]. As previously revealed by TARA Oceans metagenomes, the most prevalent groups of bacteria in the surface ocean are *Proteobacteria*, *Actinobacteria* and *Bacteroidetes* [1, 14]. We did not find any significant differences in their gene copies between polar and non-polar metagenomes, which confirms their ubiquity. For photosynthetic microbes, there appear to be geographical preferences. For instance, genes from *Cyanobacteria* were more abundant in non-polar waters whereas reads from *Chlorophytes* and *Bacillariophytes* were more abundant in the Arctic, which matches their global biogeographies [97, 98]. All other groups identified in our metagenomes including the groups of *Apicomplexa* and *Archaea* had a patchier geographical distribution.

The retrieving of MAGs from metagenomes was not always in correspondence with the abundance of reads from specific taxonomic groups. This mismatch is potentially caused by a combination of factors. Sequencing depth, read length and the quality of reads most likely play a significant role in relation to genome size and complexity. The latter two factors might be the reason why we did not retrieve any MAGs from *Apicomplexa* such as dinoflagellates. The intraphylum diversity most likely plays a role, too [99]; populations with low diversity and high coverage have been observed to improve the quality of MAGs recovered by Metabat [100]. *Viridiplantae* show low diversity, and especially members from the *Prasinophytes* have small genomes and are abundant in the surface ocean [101], which might explain why we retrieved several MAGs from different classes. Overall, completeness was lower in the eukaryotic MAGs we recovered than those MAGs reported in prior eukaryotic binning studies [12, 89]. Thus, in-depth comparative analyses using eukaryotic MAGs from the same genus or order to explore the differences in their gene composition linked to local adaptation, for instance, was not possible due to a significant number of gaps in their MAGs.

The proportion of prokaryotic MAGs recovered from different phyla is similar to those found in a larger study of oceanic genomic diversity which recovered 2631 [14]. In both, the largest number of MAGs was from proteobacteria, followed by *Bacteroidetes*. Our results did not recover MAGs from some phyla which were recovered in high numbers by [14]. For example, 167 *Chloroflexi* MAGs were recovered in [14], where none of the MAGs we recovered was identified as *Chloroflexi*. Despite appearing to be one of the more abundant phyla in our sample, neither binning effort identified *Firmicutes* MAGs, although similar studies using human gut data have [13, 14].

A recent study recovered over 700 eukaryotic genomes: 683 eukaryotic MAGs along with 30 single-amplified genomes (SAGs). The genomes total 25.2 Gbp in length with 10,207,450 predicted genes, originating from 280 billion reads from 798 samples originating from the *Tara* Oceans expeditions [19]. Although our dataset is smaller with approximately 1.5% the size in terms of reads (4.5 billion reads from 12 samples), we recovered MAGs at a similar ratio of approximately 9 billion reads per Gbp recovered, compared to 11 billion reads per Gbp recovered in the *Tara Ocean* dataset [19]. Thus, starting from a more restricted dataset, it is still possible to recover a comparable volume of MAGs as exemplified for *Bacillariophyta* MAGs (Fig. 7). Although the number and diversity of retrieved *Bacillariophyta* MAGs are higher in the *Tara Oceans* dataset, our set of MAGs is distributed over a significant number of clades. Hence, smaller metagenome studies are still providing access to uncultured genomic microbial diversity and their MAGs.

### Biogeographical distribution of MAGs and inter-kingdom species associations

**Fig. 5:**
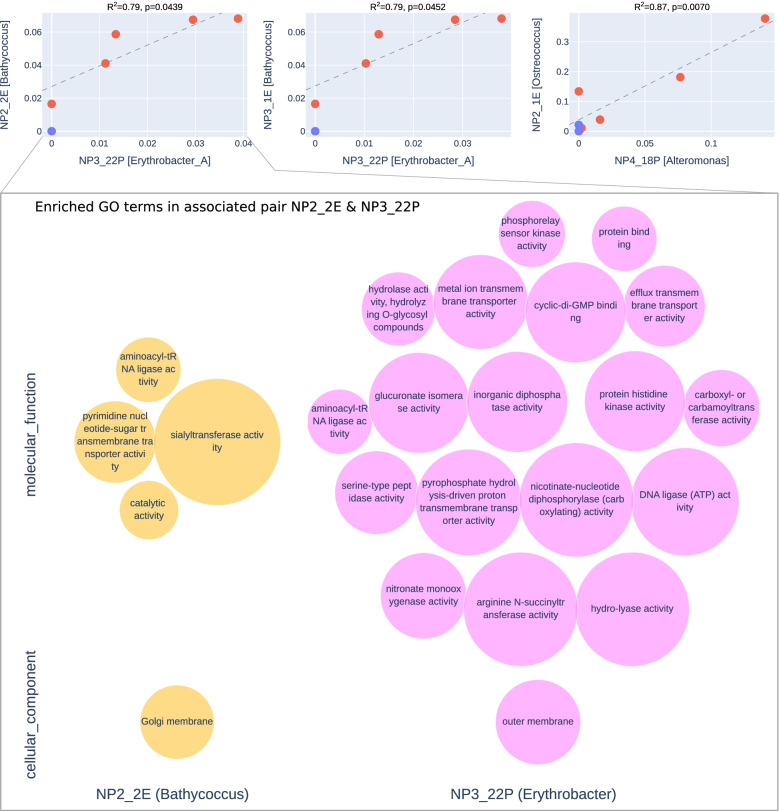
Association between eukaryotic and prokaryotic MAGs. At the top, scatter plots showing coverage of pairs of MAGs. Coverage of eukaryotes is on the vertical axis, and coverage of a prokaryotic MAG is on the horizontal axis. Each point shows coverage values in one sample. Colour of points indicates whether the point is a polar (blue) or non-polar (red) sample. Linear regression was performed with an ordinary least squares method for each pair, and regression line is shown in dashed grey. We retained pairs with *R*^2^ ≥ 0.7 and *p* ≤ 0.05 after highly influential points (outliers) were removed. Only prasinophytes are shown here; the remainder are available in Additional file 8. The lower plot shows GO terms which appear enriched in one of the associated pairs, NP2_2E and NP3_22P, a Bathycoccus and Erythrobacter, respectively. Enrichment was assessed via Fisher’s exact test detailed in the “Methods” section. Each circle represents an enriched term, and size is scaled based on the odds ratio. Left peach terms are those in the eukaryotic MAG, and right pink terms are those in the associated prokaryotic MAGs. Terms from the cellular component and molecular function GO terms are shown, divided vertically

The very pronounced demarcation between the polar Arctic and non-polar Atlantic MAGs (Fig. 4) for both prokaryotes and eukaryotes likely is a consequence of how major differences between both climatic regions have shaped the evolution and genomic diversity of phytoplankton communities [8, 102]. The most significant difference is the seasonal presence of sea ice in the Arctic Ocean. Freezing and melting of the surface ocean has a major impact on thermohaline mixing and therefore a variety of key environmental factors (e.g. light, nutrients) in addition to the overall low temperature in polar waters shaping the evolution, diversity and activity of pelagic organisms [8, 102]. It has previously been proposed that the seascape boundary between seasonally mixed and permanently stratified waters at around the 15 °C annual-mean isotherm separates global differences in oceanic primary production [102]. This isotherm also appears to be responsible for the latitudinal partitioning of microbiome compositions based on global ocean metatranscriptomes and metagenomes [8]. As this isotherm is separating our polar and non-polar communities although the polar sampling stations were further north of the 15 °C annual-mean isotherm, it is likely causative for the strong demarcation between polar and non-polar MAGs. This suggests that this ecological boundary does not only affect the distribution of individual sequences in complex meta-omics datasets but also the diversity of genomes. However, some prokaryotic MAGs (e.g. P1_16P, P1_21P, NP_23P, NP_10P) have crossed this boundary, which might indicate the presence of locally adapted ecotypes. None of the eukaryotic MAGs has been found on both sides of the boundary, which suggests that the environmental differences might have had a stronger impact on the diversification and therefore likely adaptation and evolution. These MAG-specific geographical distribution patterns are reflected in cross-kingdom co-occurrences between eukaryotes and prokaryotes in these phytoplankton communities (Fig. 5, Additional file 8). The co-occurrence patterns we identified were limited to either the Arctic or Atlantic side of the ecological boundary. Thus, none of them was crossing it, which indicates that co-evolution under significantly different environmental conditions was likely driving the formation of these associations. The GO enrichment results suggest that the identified inter-kingdom species associations are not random. For instance, enrichment of processes associated with the Golgi membrane (GO category cellular component) in the *Bathycoccus* NP2_2E MAG indicate substrate transport and secretion. The associated MAG from the heterotrophic bacterium *Erythrobacter* NP3_22P on the other hand is also enriched in membrane processes associated with transport (e.g. metal ion, proton transport, efflux transport) and metabolism involved in transforming substrates (e.g. hydrolase activity, glucuronate isomerase activity). Thus, these results suggest enrichment of exchange processes across membranes as expected for mutualistic partnerships between autotrophs and their heterotrophic partners residing in the phycosphere where organic matter released by the autotroph is used as substrates for the prokaryote in return of essential growth compounds [33, 34].

### Polar vs non-polar metabolism in MAGs

**Fig. 6:**
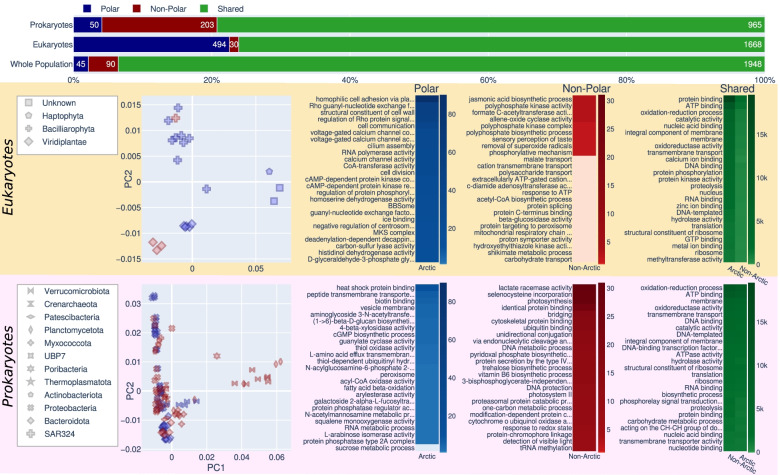
Summary of functional annotation of MAGs. In the top, each horizontal bar shows how many GO terms are found only in polar sequences (blue), found only in non-polar (red), and found in both (green). This is shown for prokaryotic MAGs, eukaryotic MAGs, and the whole population metagenome before binning. Below, the peach box shows the information for eukaryotic MAGs and the pink box for prokaryotic MAGs. For each of these, the leftmost is a PCA plot of the proportion of GO terms each MAG; each point is a MAG, with symbols showing the taxonomy of each point and colour indicating whether it was recovered from a polar or non-polar sample. To the right, heatmaps indicate the most abundant GO terms unique to polar (blue), non-polar (red), or shared (green); longer names have been truncated with ellipses

**Fig. 7:**
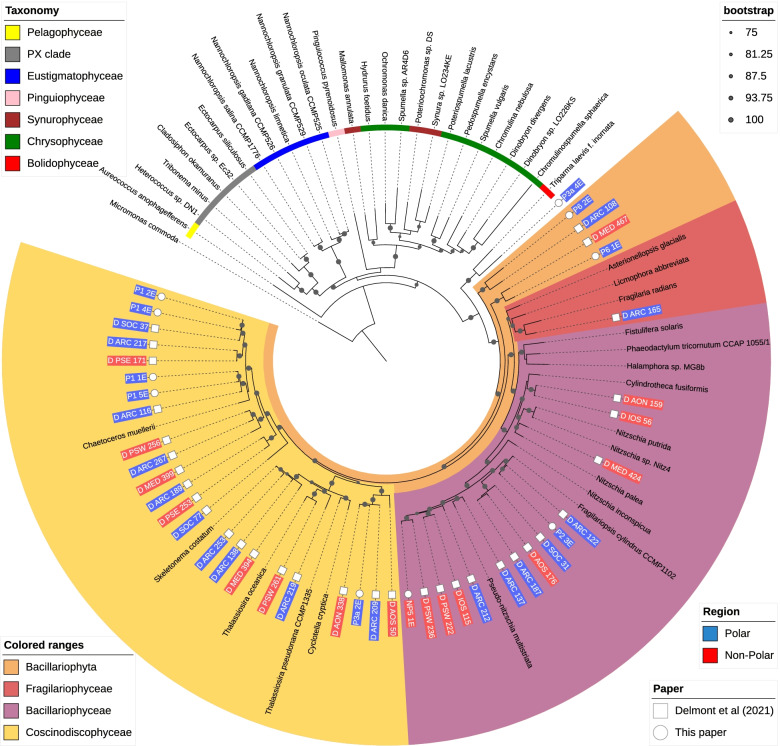
Phylogenomic tree of combining diatom MAGs from two sources. The tree was constructed using a subset of BUSCO genes. Our Bacillariophyta MAGs, along with non-redundant Bacillariophyta MAGs from Delmont et al. [19], were included, along with 18 Bacillariophyta reference genomes, 26 other Ochrophyta references, and *Micromonas commoda* as an outgroup. Leaf labels indicate whether MAGs originated from polar or non-polar data, blue and red respectively. The paper the MAGs originate from is indicated by either a square or circle. Grey dots show bootstrap values

The separation of GO terms into taxonomic groups confirms the overall taxonomic placements of the MAGs based on concatenated phylogenetic approaches, even though the GO term separation is less clear for prokaryotes (Fig. 6). The latter might be caused by a higher proportion of genetic exchange between bacterial strains compared to their eukaryotic counterparts. Although whole population metagenomics already provided some evidence that there are region-specific functions (Fig. 6), only the specific analysis of MAGs has revealed significant differences between prokaryotes and eukaryotes in terms of their genetic repertoire in relation to their geography. The reason for eukaryotic MAGs to have more unique Pfams in polar waters of the Arctic and vice versa for prokaryotes remains enigmatic but suggests that identical environmental conditions and therefore similar selection pressures would impose differences in how prokaryotic and eukaryotic genomes evolve in the surface ocean. It appears that for eukaryotes, a dynamic surface ocean with seasonal mixing and sea-ice formation requires genomes to diversify because of the high abundance of transposable elements [35]. In contrast, prokaryotic MAGs in the same environment were characterised by a high abundance of domains of unknown function. Non-polar environments characterised by higher temperatures, stratified waters and weaker seasonality appear to enrich for PSD domains that are shared by chytochrome c-like proteins for electron transport as part of the respiratory chain in prokaryotes (Fig. 6). This potentially suggests that respiratory activity is enhanced in non-polar prokaryotes compared to their polar counterparts, which would be expected according to the positive relationship between temperature and metabolic activity [103]. Interestingly, Pfams related to phosphate acquisition and metabolism in addition to Pfams involved in iron metabolism and electron transport were among the most enriched domains in non-polar eukaryotic MAGs (Fig. 7). The relatively low nutrient concentrations in these stratified waters might only allow eukaryotes to thrive if they have developed mechanisms for the efficient uptake of nutrients [104, 105]. Smaller-sized prokaryotes with streamlined genomes usually outcompete eukaryotes in these environments as their nutrient demand is lower [104].

## Conclusions

Our study has revealed that significant differences in environmental conditions of the surface ocean (e.g. cold and mixed vs warm and stratified waters) do not only impact the diversity of prokaryotic and eukaryotic genomes but also their inter-kingdom species associations, which has not been studied before using MAGs. Thus, MAG-based analyses of phytoplankton microbiomes not only offer the identification of novel genomic resources, they might reveal unifying concepts responsible for how differences in ecosystem properties shape the genomes of their inhabitants and even species associations, which underpin the evolution of complex microbial communities.

## Supplementary Information

**Additional file 1.** Summary details of data, including environmental metadata for each sample. Project identifiers for accessing data and analysis on the IMG website are provided. Includes worksheets giving completeness and contamination of MAGs.

**Additional file 2.** Average Nucleotide Identity plots and data in tab-separated format for related groups of MAGs and reference genomes.

**Additional file 3.** Amino Acid Identity plots and data in tab-separated format for related groups of MAGs and reference genomes.

**Additional file 4.** Table summarising each assembled sample, prior to binning. Includes tables in comma separated format giving estimated number of genes copies in which each Pfam domain or GO term occurs, as well as PCA plot of the samples based on the Pfam data.

**Additional file 5.** Includes a table giving the percentage of estimated gene copies by taxonomy, and a box plot showing distribution of relative abundance for each domain, split up between polar and non-polar stations.

**Additional file 6.** Tree distances between MAGs and the closest Polar and Non-Polar MAGs, displayed as box plots. Statistics between pairs are p-values from Mood’s median test for difference in sample medians.

**Additional file 7.** For eukaryotes and prokaryotes, phylogenomic tree in Newick format, list of the Phylosift marker genes included when building tree, and details of reference genomes included in tab-separated format.

**Additional file 8.** Additional scatter plots showing normalised coverage at stations between polar eukaryotic and prokaryotic MAGs where some association was observed. Axes show coverage per million reads. Vertical axes are coverage for eukaryotic MAG, horizontal shows coverage for prokaryote. Each row of plots shows associations with one eukaryotic MAG.

**Additional file 9.** Mean coverage of each MAG in the reads for each sample, provided in comma separated format.

**Additional file 10.** Results of enrichments on two controls sets of MAGs which did not participate in cross domain associations. Plots show the number of enriched terms shared with the pair discussed in the body of the text.

## Data Availability

We have made available via FigShare some additional files referred to in the paper, along with MAGs, functional annotations and BLAST-based taxonomic analysis generated and during the current study in the “Metagenome-assembled genomes of phytoplankton communities across the Arctic Circle repository” repository, 10.6084/m9.figshare.c.5017517 [106]. The metagenomic sequencing reads and assemblies analysed during the current study are available on the JGI Genome Portal repository PhycoCosm, using the project identifiers provided in Additional file 1, https://genome.jgi.doe.gov/ [107, 108].
