# Supplementary figures and images for: Metagenome-assembled genomes of phytoplankton microbiomes from the Arctic and Atlantic Oceans
Source: Microbiome. 2022 Apr 28;10:67. doi: 10.1186/s40168-022-01254-7 (PMC9047304; doi:10.1186/s40168-022-01254-7)

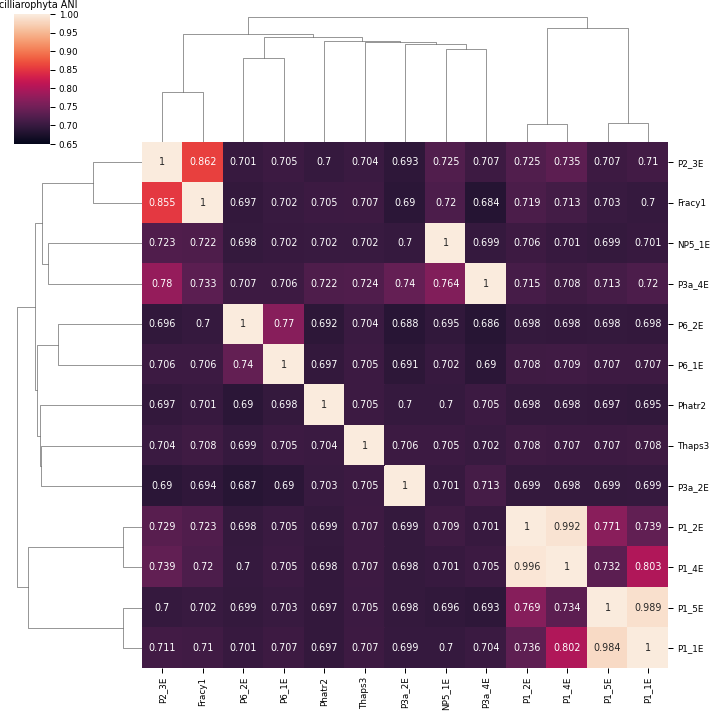

Supplement: Supplementary file 3 — Additional file 2. Average Nucleotide Identity plots and data in tab-separated format for related groups of MAGs and reference genomes. [file 40168_2022_1254_MOESM3_ESM.gz › 2. ani/Bacilliarophyta_ANI.png]

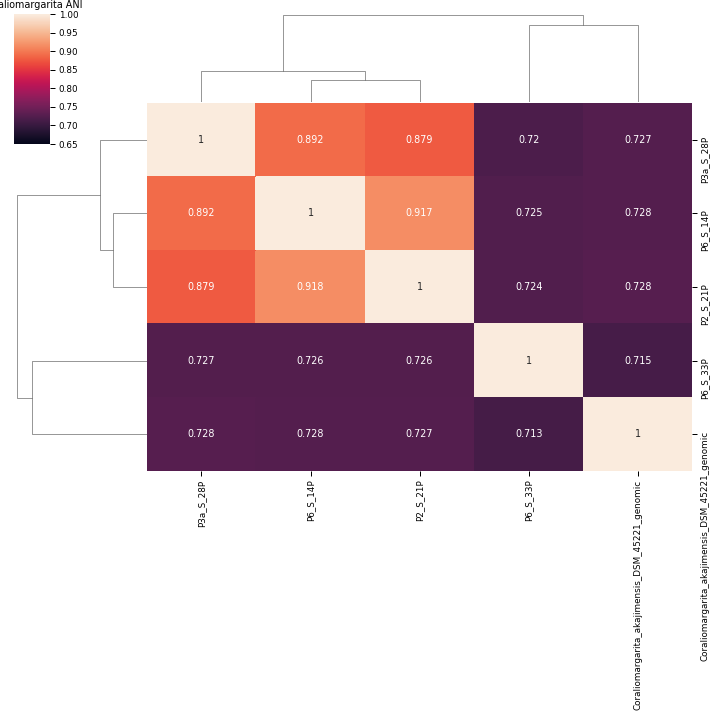

Supplement: Supplementary file 3 — Additional file 2. Average Nucleotide Identity plots and data in tab-separated format for related groups of MAGs and reference genomes. [file 40168_2022_1254_MOESM3_ESM.gz › 2. ani/coraliomargarita_ani.png]

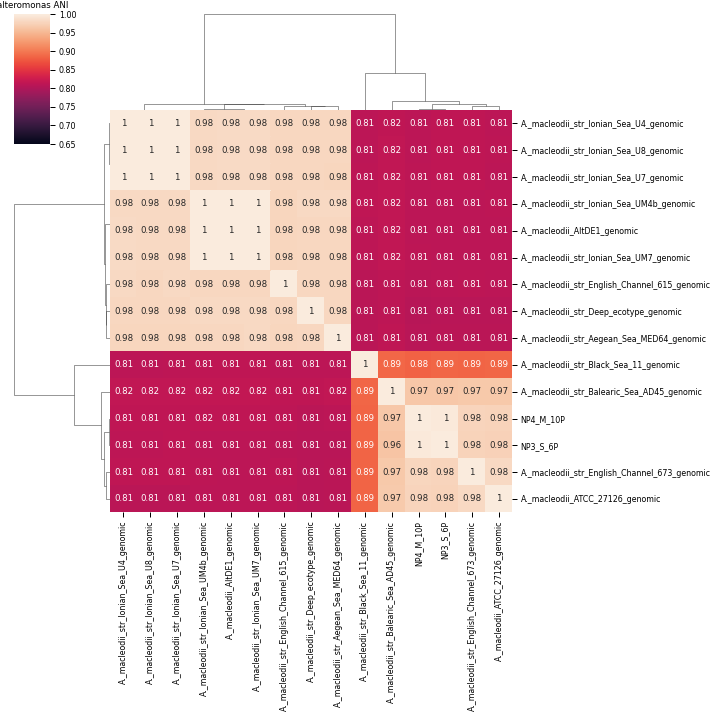

Supplement: Supplementary file 3 — Additional file 2. Average Nucleotide Identity plots and data in tab-separated format for related groups of MAGs and reference genomes. [file 40168_2022_1254_MOESM3_ESM.gz › 2. ani/alteromonas_ani.png]

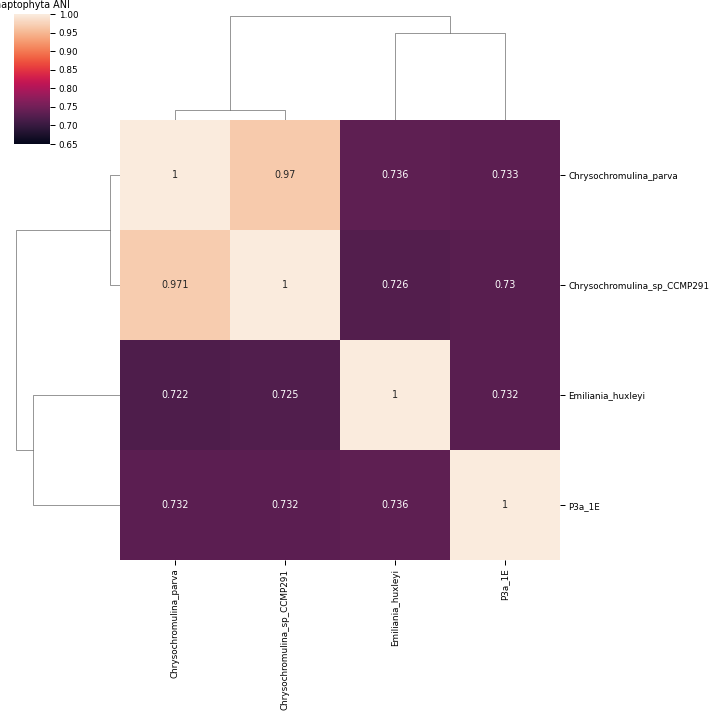

Supplement: Supplementary file 3 — Additional file 2. Average Nucleotide Identity plots and data in tab-separated format for related groups of MAGs and reference genomes. [file 40168_2022_1254_MOESM3_ESM.gz › 2. ani/Haptophyta_ANI.png]

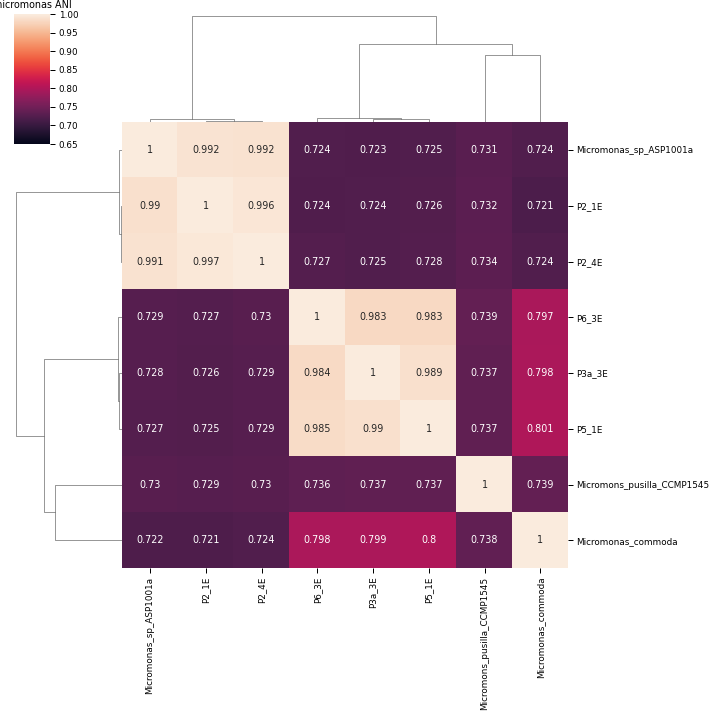

Supplement: Supplementary file 3 — Additional file 2. Average Nucleotide Identity plots and data in tab-separated format for related groups of MAGs and reference genomes. [file 40168_2022_1254_MOESM3_ESM.gz › 2. ani/Micromonas_ANI.png]

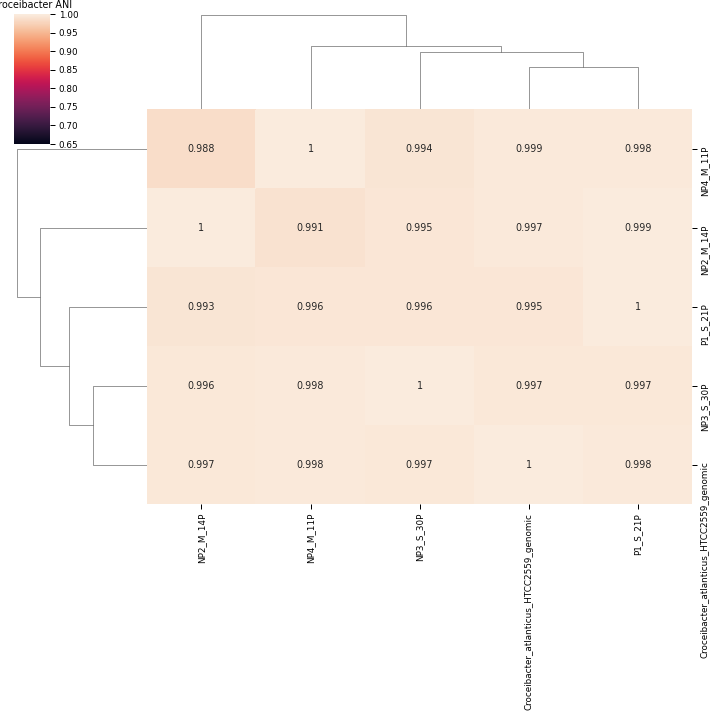

Supplement: Supplementary file 3 — Additional file 2. Average Nucleotide Identity plots and data in tab-separated format for related groups of MAGs and reference genomes. [file 40168_2022_1254_MOESM3_ESM.gz › 2. ani/croceibacter_ani.png]

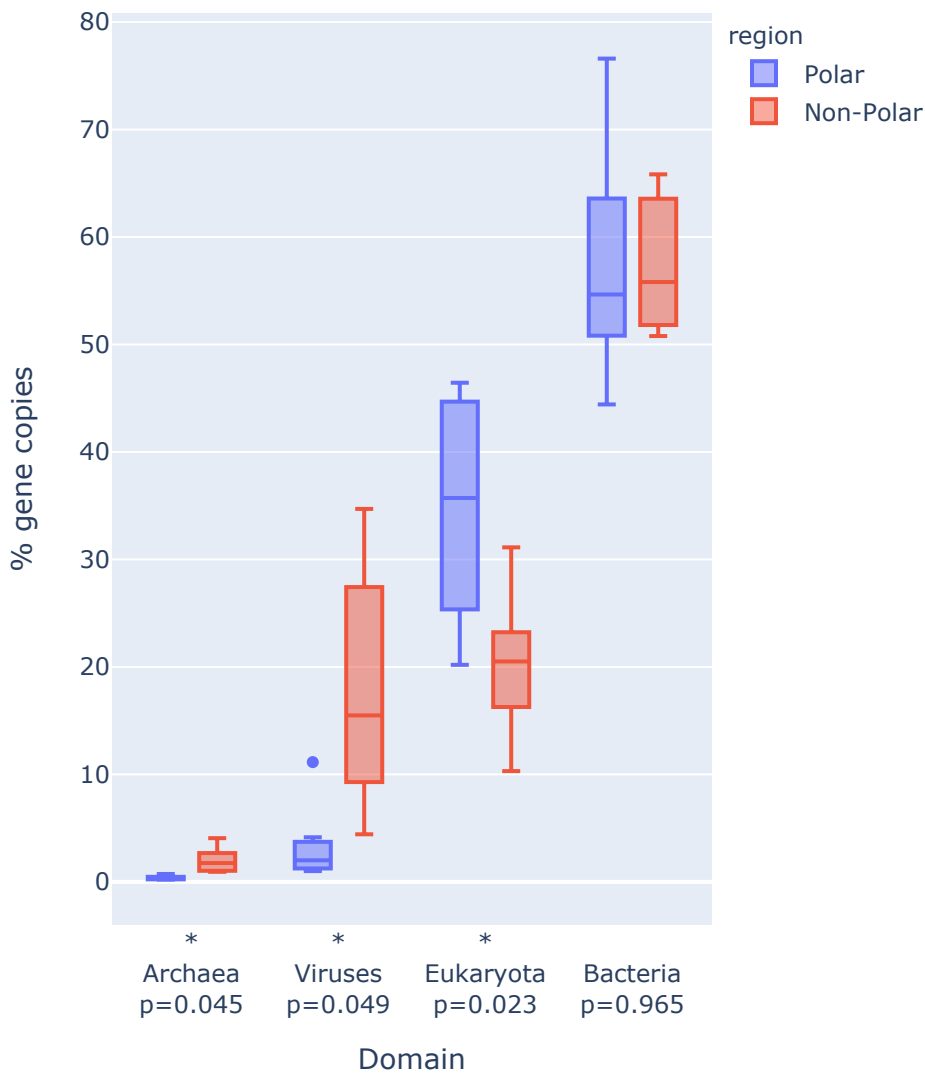

Supplement: Supplementary file 6 — Additional file 5. Includes a table giving the percentage of estimated gene copies by taxonomy, and a box plot showing distribution of relative abundance for each domain, split up between polar and non-polar stations. [file 40168_2022_1254_MOESM6_ESM.gz › 5. taxonomy/taxonomy_genecopies_boxplot.pdf]

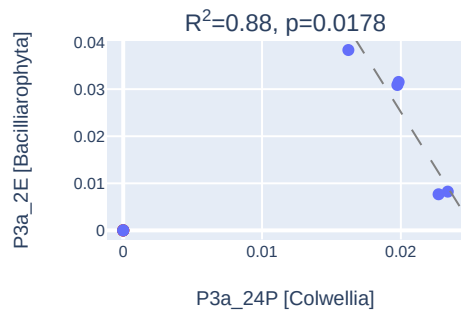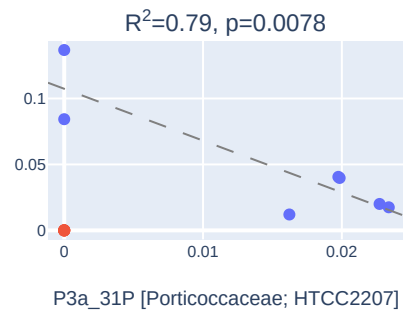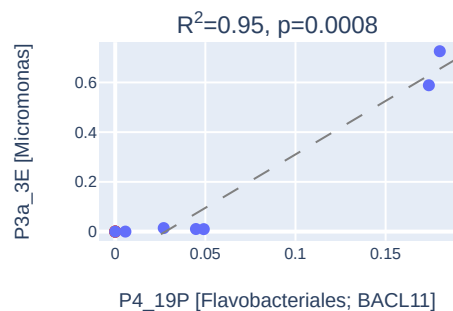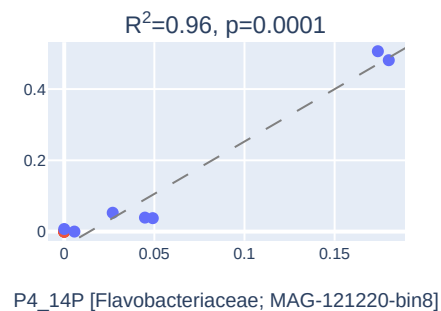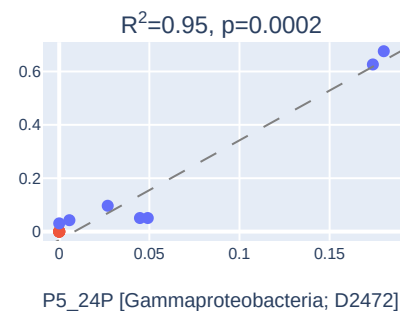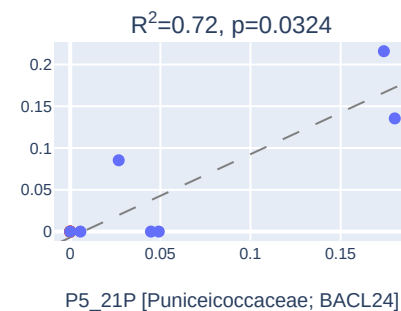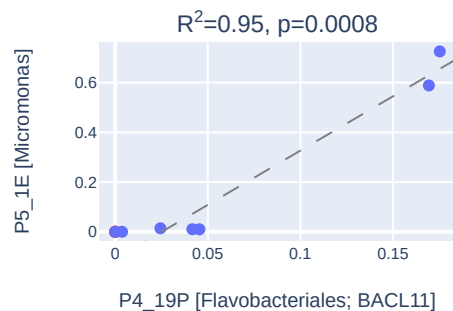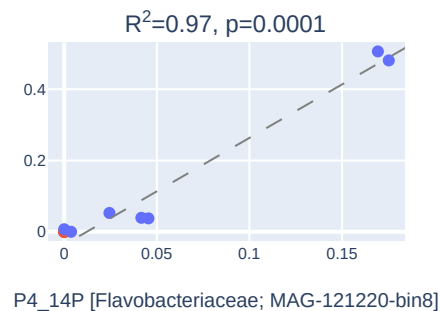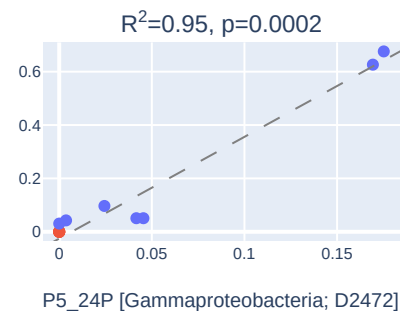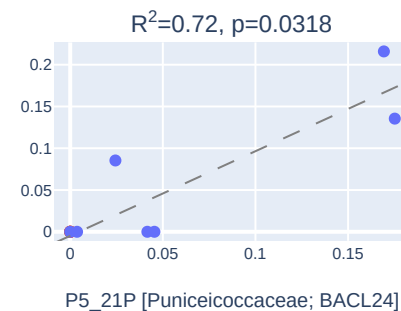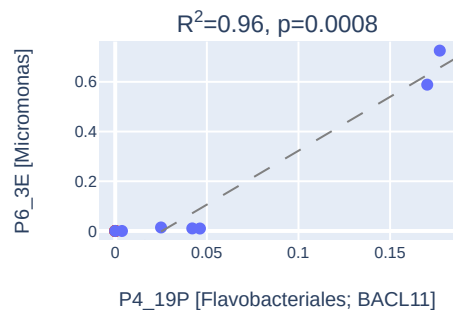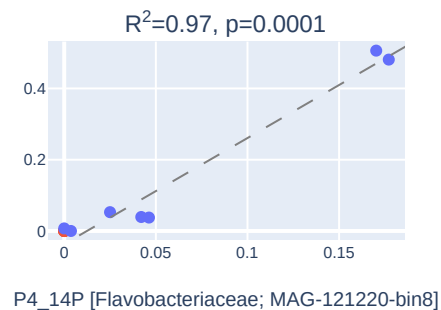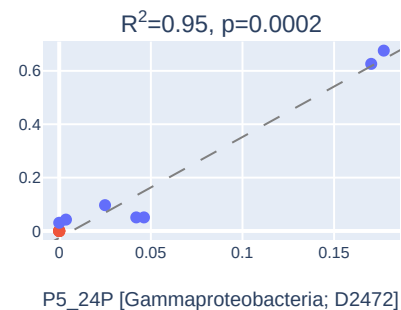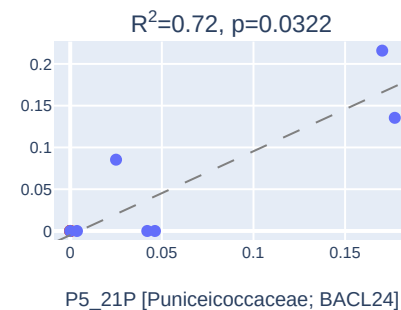

Supplement: Supplementary file 9 — Additional file 8. Additional scatter plots showing normalised coverage at stations between polar eukaryotic and prokaryotic MAGs where some association was observed. Axes show coverage per million reads. Vertical axes are coverage for eukaryotic MAG, horizontal shows coverage for prokaryote. Each row of plots shows associations with one eukaryotic MAG. [file 40168_2022_1254_MOESM9_ESM.pdf]
