# Supplementary material for: Metagenome-assembled genomes of phytoplankton microbiomes from the Arctic and Atlantic Oceans
Source: Microbiome. 2022 Apr 28;10:67. doi: 10.1186/s40168-022-01254-7 (PMC9047304; doi:10.1186/s40168-022-01254-7)
Supplement: Supplementary file 5 — Additional file 4. Table summarising each assembled sample, prior to binning. Includes tables in comma separated format giving estimated number of genes copies in which each Pfam domain or GO term occurs, as well as PCA plot of the samples based on the Pfam data. [file 40168_2022_1254_MOESM5_ESM.gz › 4. population function/population function pca.pdf]

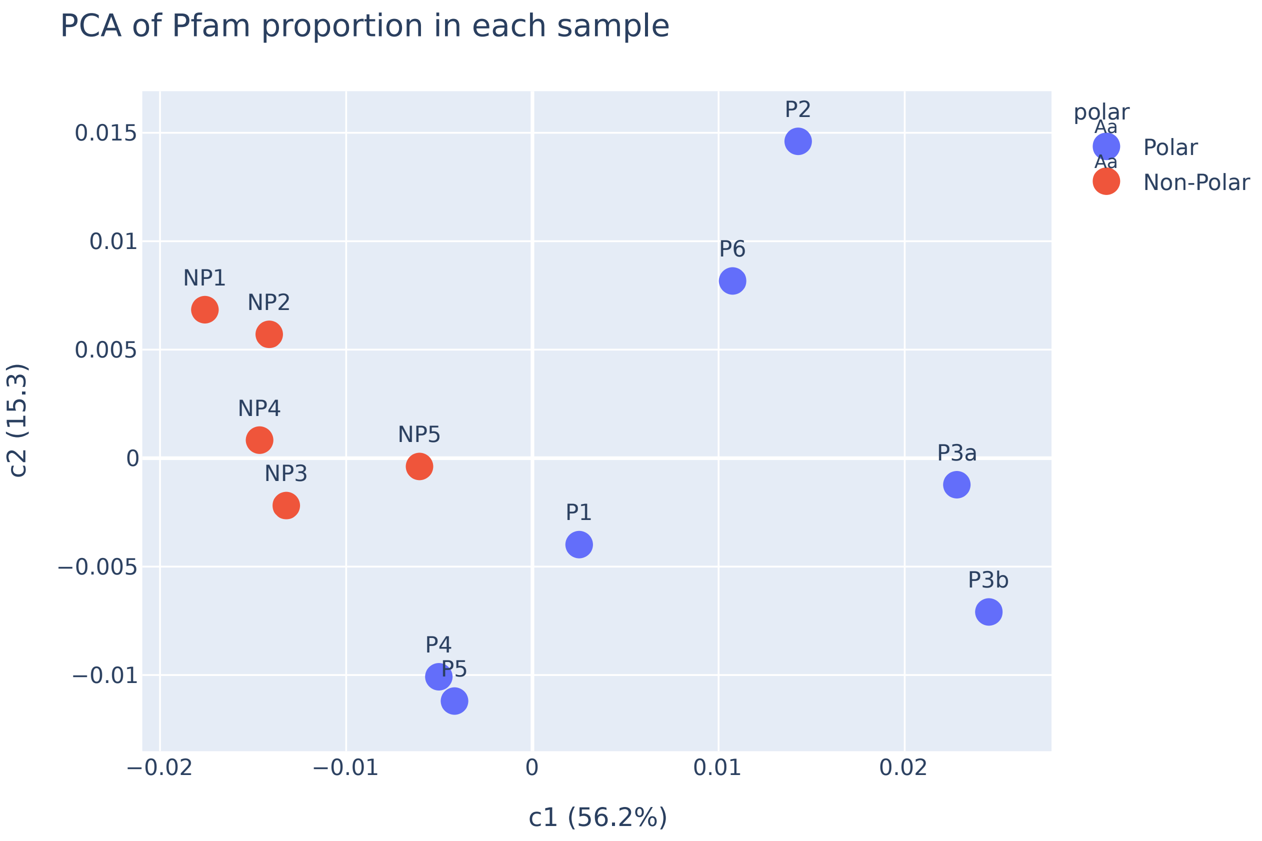

Principal component analysis was performed on the proportion of Pfam domains observed in assembled contigs in each sample, before any binning was performed. The horizontal axis shows the first axis of the decomposition, the vertical the second axis. Each axis is labelled with the percentage of variance which it explains. Polar stations are indicated with blue points, non-polar stations with red.

Genes were predicted from assembled contigs. The number of gene copies in the sample was estimated from the mean coverage of the contig when mapping reads back to the assembly. Each domain was assigned the sum of estimated gene copies in which that domain occurs.

This data is provided in comma separated format. Additionally, the same data mapped to GO terms is provided in the same format. We used the pfam2go mapping maintained by the Interpro team (http://current.geneontology.org/ontology/external2go/pfam2go)
