# Supplementary material for: Metagenome-assembled genomes of phytoplankton microbiomes from the Arctic and Atlantic Oceans
Source: Microbiome. 2022 Apr 28;10:67. doi: 10.1186/s40168-022-01254-7 (PMC9047304; doi:10.1186/s40168-022-01254-7)
Supplement: Supplementary file 7 — Additional file 6. Tree distances between MAGs and the closest Polar and Non-Polar MAGs, displayed as box plots. Statistics between pairs are p-values from Mood’s median test for difference in sample medians. [file 40168_2022_1254_MOESM7_ESM.pdf]

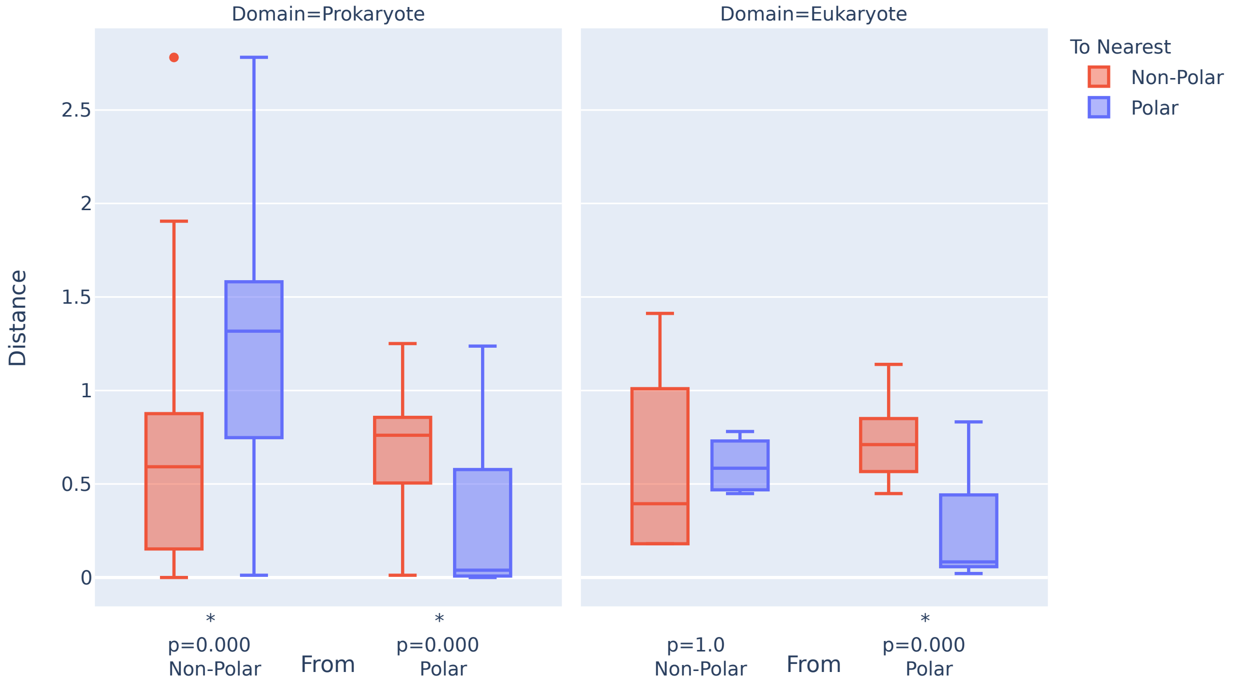

Distribution of the distance from each MAG to the near Polar or Non-Polar MAG. The MAG being measured from is split on the x-axis, the distance to the nearest MAG from either Polar or Non-Polar is indicated by colour. Difference in median between distances to Polar/Non-Polar assessed using Mood's median test, p-values given on x-axis.
