# Supplementary material for: Metagenome-assembled genomes of phytoplankton microbiomes from the Arctic and Atlantic Oceans
Source: Microbiome. 2022 Apr 28;10:67. doi: 10.1186/s40168-022-01254-7 (PMC9047304; doi:10.1186/s40168-022-01254-7)
Supplement: Supplementary file 11 — Additional file 10. Results of enrichments on two controls sets of MAGs which did not participate in cross domain associations. Plots show the number of enriched terms shared with the pair discussed in the body of the text. [file 40168_2022_1254_MOESM11_ESM.pdf]

Enriched GO terms for non-associated MAGs

Following the same method given in the main text, we look at what terms appear enriched for some control MAGs which did not participate in the Eukaryote/Prokaryote associations identified from mean coverage. The intersection between terms enriched in these control MAGs and the two associated MAGs is shown using venn diagrams. Our intention was to investigate whether the same sets of terms would appear enriched regardless, or whether they are specific to the associated MAG.

We assessed enrichment using Fisher's exact test. For input data we summed the number of times each term was observed in the background set of MAGs (*B*), and the number of times each term was observed in a selected MAG (*S*). For testing an individual term (*T*), the contingency table was therefore in the form:

	in *S*	in *B*
Count of term *T*	13	13
Count of other terms not *T*	56	1008

Using a one-sided test we looked for terms which were positively associated with *S*, with a threshold of $p \leq 0.05$.

For testing enrichment of eukaryotes, our background set B_e consisted of all MAGs we identified as prasinophytes, which were not in any of the identified associations. This consisted of two MAGs. If MAG *S* was also a prasinophyte, we removed it from B_e for those tests.

In the case of prokaryotes, background set B_p consisted of all MAGs identifed as alphaproteobacteria, which were not in any of the identified associations. This consisted of 12 MAGs Again where *S* was also an alphaproteobacteria, we removed it from B_p for those tests.

We selected two pairs of MAGs, each one consisting of one eukaryote and one prokaryote. The first pair is more distantly related to the background sets, the second more closely related.

Distant pair: NP3a_4E (Bacillariophyta) and NP3_6P (Gammaproteobacteria)

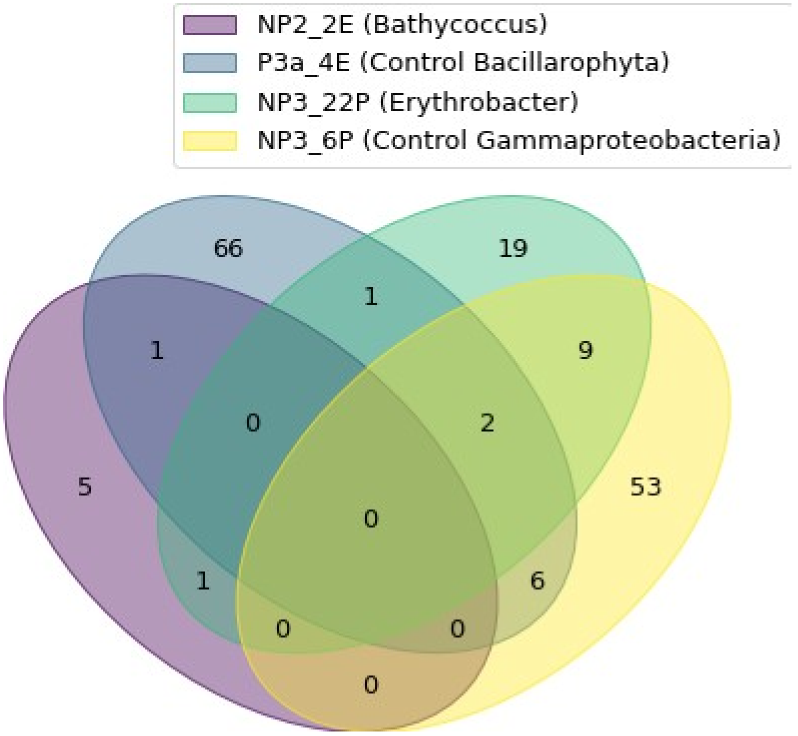

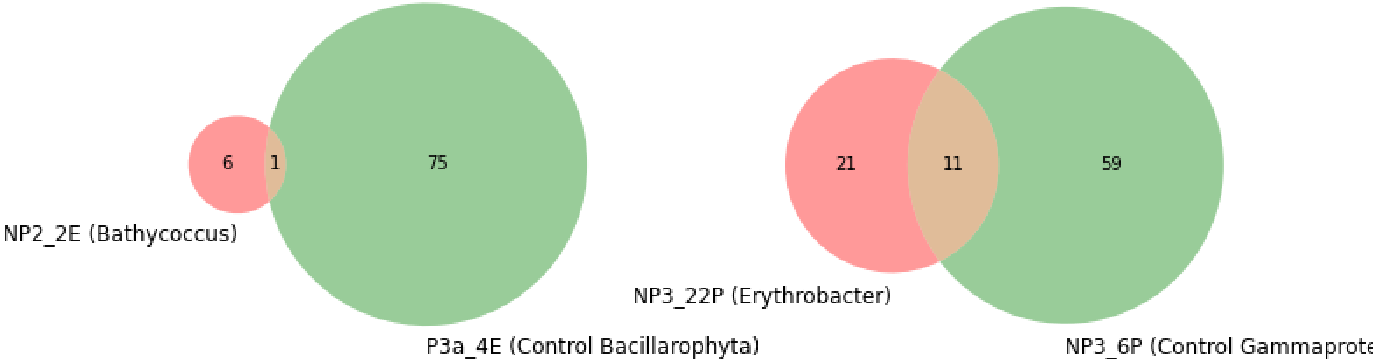

Many more terms in the bacillariophyta and gammaproteobacteria MAGs were enriched, which could be explained by the taxonomic difference from the background sets of prasinophytes and alphaproteobacteria. The only term shared between the eukaryotes is a high level one (GO:0003824 catalytic activity).

Close pair: P2_1E (Prasinophyte) and NP3_6P (Alphaproteobacteria)

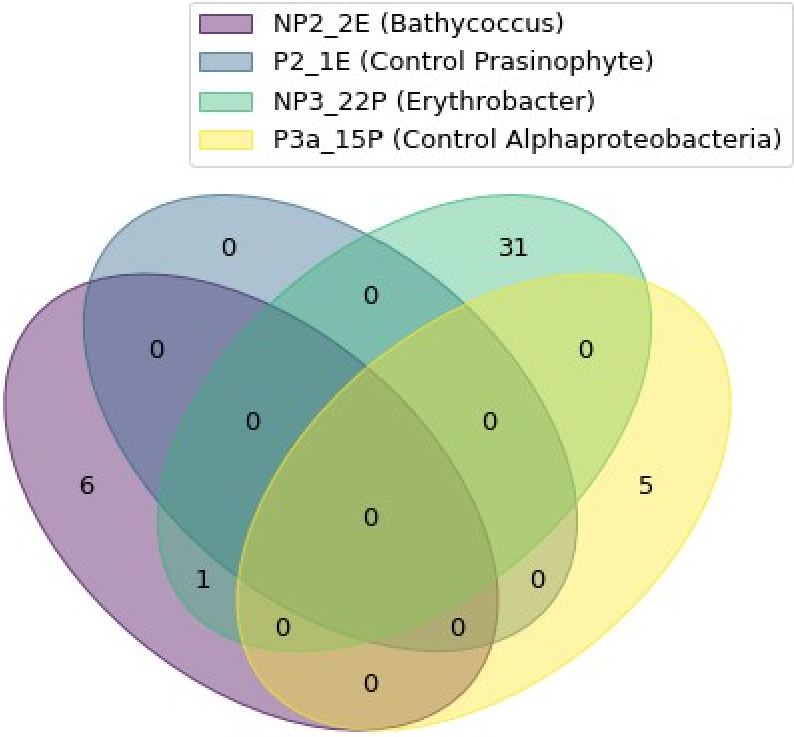

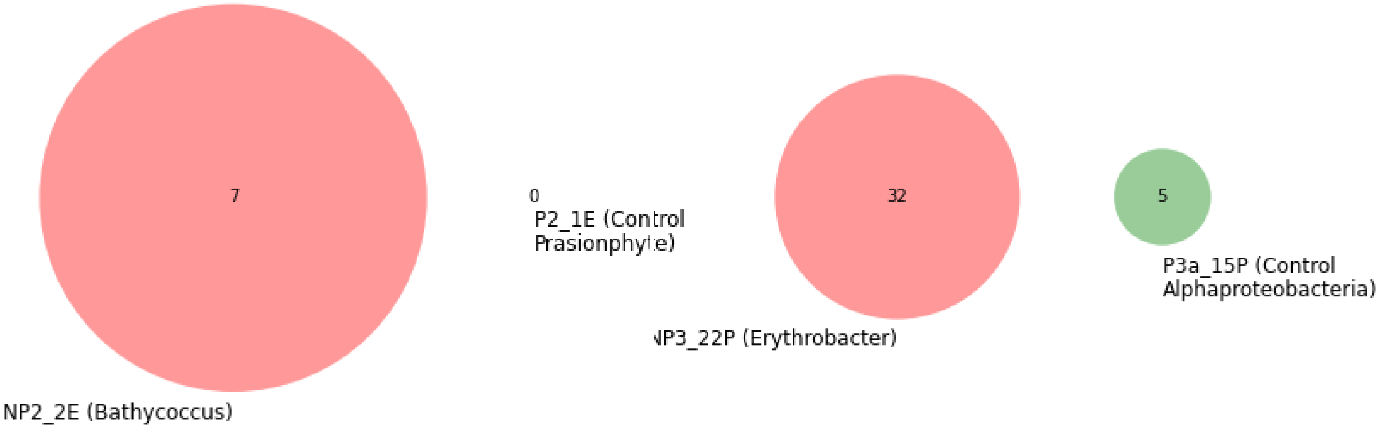

No terms appeared enriched in our selected control prasinophyte P2_1E. For these tests we were comparing organisms which appear very taxonomically similar so a lack of divergence in function is unsurprising; background set B_p consisted of only one MAG, which is very closely related to P2_1E in our phylogenomic tree. There we no enriched terms shared by the prokaryotes, suggesting the enriched terms for NP3_22P could be specific to association identified between it and NP2_2E.
